# The antidiabetic drug metformin acts on the bone microenvironment to promote myeloma cell adhesion to preosteoblasts and increase myeloma tumour burden *in vivo*

**DOI:** 10.1016/j.tranon.2021.101301

**Published:** 2021-12-08

**Authors:** Beatriz Gámez, Emma V. Morris, Sam W.Z. Olechnowicz, Siobhan Webb, James R. Edwards, Aneka Sowman, Christina J. Turner, Claire M. Edwards

**Affiliations:** a Nuffield Department of Surgical Sciences, University of Oxford, Oxford, UK; b Oxford Centre for Translational Myeloma Research, University of Oxford, Oxford, UK; c Nuffield Department of Orthopaedics, Rheumatology and Musculoskeletal Sciences, University of Oxford, Oxford, UK

**Keywords:** Myeloma, Metformin, Osteopontin, Osteoblast, Bone microenvironment

## Abstract

Multiple myeloma is a haematological malignancy that is dependent upon interactions within the bone microenvironment to drive tumour growth and osteolytic bone disease. Metformin is an anti-diabetic drug that has attracted attention due to its direct antitumor effects, including anti-myeloma properties. However, the impact of the bone microenvironment on the response to metformin in myeloma is unknown. We have employed *in vitro* and *in vivo* models to dissect out the direct effects of metformin in bone and the subsequent indirect myeloma response. We demonstrate how metformin treatment of preosteoblasts increases myeloma cell attachment. Metformin-treated preosteoblasts increased osteopontin (OPN) expression that upon silencing, reduced subsequent myeloma cell adherence. Proliferation markers were reduced in myeloma cells cocultured with metformin-treated preosteoblasts. *In vivo*, mice were treated with metformin for 4 weeks prior to inoculation of 5TGM1 myeloma cells. Metformin-pretreated mice had an increase in tumour burden, associated with an increase in osteolytic bone lesions and elevated OPN expression in the bone marrow. Collectively, we show that metformin increases OPN expression in preosteoblasts, increasing myeloma cell adherence. *In vivo*, this translates to an unexpected indirect pro-tumourigenic effect of metformin, highlighting the importance of the interdependence between myeloma cells and cells of the bone microenvironment.

## Highlights

• Metformin has unexpected indirect effects on the bone microenvironment to promote myeloma.
• Pretreatment with metformin indirectly increases myeloma tumour burden *in vivo*.
• Metformin increases myeloma cell adhesion to osteoblasts via osteopontin.

## Introduction

Multiple myeloma is a B-cell malignancy characterised by an expansion of clonal plasma cells within the bone marrow. This colonization leads to bone marrow failure, anaemia and the characteristic osteolytic bone lesions associated with disease progression [1]. Multiple myeloma remains an invariably fatal haematological malignancy and is preceded by the benign condition monoclonal gammopathy of undetermined significance (MGUS) [2]. MGUS patients show early infiltration of plasma cells but no evidence of end-organ damage. The time between diagnosis of MGUS and progression to myeloma can vary widely, with a median of 10.4 years and so there is an extensive period for potential clinical management and an increasing interest in preventative measures [3], [4], [5].

Metformin has been used for many years to improve glycaemic control in patients with type 2 diabetes. While the primary function of metformin is to decrease blood glucose levels by reducing hepatic glucose production, the exact molecular mechanism is not well understood. Recently, metformin has gained attention due to its direct antitumor activity in a number of cancers, including multiple myeloma. Clinically, metformin use is associated with improved outcomes in patients with myeloma and epidemiological studies have demonstrated an association between metformin use and reduced transformation from MGUS to multiple myeloma [6], [7], [8], [9], [10], [11]. A retrospective study of U.S veterans with diabetes mellitus prior to MGUS diagnosis identified a reduction in MGUS progression to myeloma when metformin was consistently used for at least 4 years [12]. There is accumulating *in vitro* and *in vivo* evidence for a direct anti-tumour effect of metformin in myeloma, however, preclinical *in vivo* studies were limited to subcutaneous models and the critical influence of the bone marrow microenvironment on both tumour growth and osteolytic bone disease was not fully considered [13], [14], [15], [16], [17], [18], [19], [20].

The impact of metformin within bone is unclear, with recent research producing conflicting results [21]. Several studies have been published presenting an anabolic effect of metformin on bone [22], [23], [24], [25]. *In vitro* studies have shown an enhancement in osteoblast differentiation after metformin treatment as well as an increase in the expression of important osteoblast markers including collagen-I, osteocalcin, Runx2 or ALP [22,23,26]. In addition, metformin has been proven to inhibit formation of reactive oxygen species (ROS) and apoptosis in osteoblastic cultures exposed to high glucose concentrations [27]. *In vivo* studies have also demonstrated an increase in bone mineral density and bone microarchitecture, with results showing the ability of metformin to reduce bone loss in a number of disease states [28,29]. Studies of the effect of metformin on osteoclasts are limited, however metformin has been shown to increase circulating osteoclast precursors in postmenopausal women with type II diabetes [30]. Together these studies suggest a potential protective effect of metformin on bone health. However, in contrast, a number of studies found no osteogenic effects of metformin, with no effects on fracture healing [31], [32], [33], [34]. These contradictory results likely reflect the complex mechanism of action of metformin combined with different *in vitro* and *in vivo* disease models yet highlight the potential for metformin to directly impact the bone microenvironment.

Multiple myeloma is exquisitely dependent upon cellular interactions within the bone microenvironment to support both tumour growth and survival and the development of the osteolytic bone disease [35]. The discovery that myeloma cells home to osteoblastic haematopoietic stem cell niches places osteoblasts as major regulators of the tumour bone niche [36,37]. While at later stages of disease osteoblasts are suppressed, contributing to myeloma bone disease, at early stages osteoblasts are known to support myeloma cell homing, dormancy and disease progression. As such, the reported osteogenic effects of metformin have the potential to impact the overall response of myeloma to treatment with metformin. The aim of this study was to determine whether metformin has direct effects on the bone microenvironment that subsequently impact myeloma development and progression. Using *in vitro* and *in vivo* pre-treatment myeloma-bone model systems allowing us to separate direct and indirect effects of metformin, we demonstrate the unforeseen effects of metformin to promote myeloma cell adhesion and tumour burden via altering the bone microenvironment.

## Methods

### Metformin treatment *in vivo*

All procedures were conducted in accordance with the Animals Scientific Procedures Act of 1986 (UK) and approved by the University of Oxford Animal Welfare and Ethical Review Body (Home Office Project License 30/2996 and PCCCC8952). For *in vivo* studies, weight-matched, 5,6 week old female C57BL/KaLwRij were used (Harlan Netherlands B.V.). For specific experiments males were also used as indicated in figures legends. Within each study, mice were age, sex and weight matched between treatments. Mice were housed under standard housing, husbandry and diet conditions. Mice were randomly allocated to experimental groups. For pretreatment with metformin, mice were treated for 4 weeks prior to tumour inoculation with 2.5 mg/ml metformin in the drinking water. Metformin was then removed and mice were inoculated via tail injection with 1.5 × 10^6^ 5TGM1-GFP cells. Mice were sacrificed at day 25 post-inoculation. Bone marrow and spleen cell suspensions were filtered (70 µm filter) and analysed for percentage GFP fluorescence by flow cytometry. Serum IgG2b concentration was quantified by ELISA (Mouse IgG2b ELISa quantification Set, Bethyl Laboratories). OPN, RANKL and OPG concentrations were quantified in bone marrow and serum using ELISAs (R&D Systems). Glucose measurements were analysed using Accu-chek performa nano glucose metre (Roche). Right tibiae were formalin-fixed and microCT (Skyscan 1172 X-ray Microtomograph; Bruker MicroCT, Kontich, Belgium) performed at 37 kV/228 μA, with an isometric resolution of 9.94 μm/ pixel using a 0.5 mm aluminium filter. Reconstruction of the original scan data was performed using NRecon. The same threshold setting for bone tissue was used for all samples. On the 3D reconstructed image, osteolytic lesions on the curved medial tibial surface that completely penetrated the cortical bone and were greater than 100 μm in diameter were counted.

### Cell culture

5TGM1 murine myeloma cells were a kind gift from Prof. Gregory Mundy, University of Texas Health Science centre at San Antonio [38]. MM.1S (ATCC CRL-2974) cell lines were a gift of Prof. Udo Oppermann, University of Oxford. 2T3 mouse preosteoblasts were a kind gift from Dr. Steve Harris, University of Texas Health Science centre at San Antonio [39]. ST2 BMSCs were purchased from Riken cell Bank, MC3T3-E1 were obtained from ACACC and HS5 human stromal cell line and MG63 osteosarcoma cells were obtained from ATCC (CRL-11,882 and CRL-1427, respectively). All cell lines tested negative for mycoplasma. 5TGM1-GFP and MM.1S-GFP cells were cultured in RPMI-1640 medium supplemented with 10% foetal bovine serum (FBS), 1% L-glutamine (G7513, Sigma), Penicilin/Streptomycin (P/S, 15,140-122, Gibco), MEM non-essential amino acids (M7145, Sigma) and sodium pyruvate (S8636, Sigma). ST2 and 2T3 cell lines were cultured in DMEM with 10% foetal bovine serum (FBS), 1% L-glutamine and P/S. MC3T3-E1 cells were cultured in alpha MEM with 10% FBS, 1% Pyruvate and P/S. Metformin (317,240, Calbiochem) was used at indicated doses. Stock was prepared dissolving 5 g of metformin in 30 ml of water.

For coculture experiments, confluent 2T3 preosteoblasts or ST2 bone marrow stromal cells were treated with up to 10 mM metformin for 48 h. Culture media was then removed, cells were washed once with culture media and then 2 × 10^6^ GFP positive myeloma cells (MM1.S or 5TGM1) were plated onto the preosteoblasts or stromal cells. Following coculture, myeloma cells were imaged using a Nikon Eclipse TE300 inverted microscope. The relative proportion of myeloma cells was determined by quantification of the area of GFP-positive cells, using Image J. When indicated, an Incucyte® was used, with the proportion of GFP-positive myeloma cells quantified using Incucyte® analysis software following the recommended protocol.

### Proliferation and viability assays

Vybrant Dil cell labelling solution and CellTrace^TM^ Violet cell proliferation kit were used for proliferation assays (V22885 and C34557, Thermo Fisher). GFP-myeloma cells were stained following manufacturer's protocol on day 0 before seeding them as single culture or cocultured with 2T3. Some cells were used for flow cytometry to determine the highest level of stain at day 0. After 4 days of culture, cells were collected and studied again by FACS in order to determine the level of staining [37].

Cell viability was assessed using Alamar Blue (0.1 mg/ml). Quantification was performed on a BMG labtech FLUOstar plate reader.

### Osteoblast mineralisation

2T3 cells were seeded and osteogenic media was used for 20–21 days. Osteogenic media was prepared using alpha MEM and including 50ug/ml ascorbic acid and 20 mM β-glicerophosphate. Media was made fresh every week and changed every 2-3 days. For mineralisation assay and quantification, a 2% alizarin red staining solution was prepared using water. Cells were fixed for 10 min with 10% formalin, washed with milliQ water and then alizarin red was added for 30 min. Nikon Eclipse TE300 inverted microscope was used to image 3–5 fields per well. Image J was used for quantification.

### siRNA experiments

Osteopontin siRNA, control siRNA and control siRNA-fluorescein conjugate were used for transient transfection (sc-36,130, sc-37,007, sc-36,869, Santa Cruz Biotechnology). 4 µl of RNAiMAX lipofectamine (13,778,075, Thermo Fisher) and 30 pmol siRNA were used per 6-well. In order to increase levels of OPN repression in some longer experiments, cells were seeded, next day transfected and retransfected again 48 h later when also metformin was added. Then media was removed and myeloma cells were seeded on top (direct coculture). Nikon Eclipse TE300 inverted microscope was used to image 3–5 GFP fields per well. Image J was used for quantification.

### RT-qPCR

RNA isolation was carried out using RNaeasy mini kit (74,104, QIAGEN) following manufacturer's protocol. RNA was then reverse transcribed using Precision DNase kit (DNASE-50, Primerdesign) and Precision nanoScript2 Reverse Transcription Kit (RT-NanoScript2, Primerdesign). Sybr Green qPCR was performed using Fast SYBR^TM^ Green Master mix (Thermo Fisher). The following primers were used: Mm OPN (F:AGCCACAAGTTTCACAGCCACAAGG, R:CTGAGAAATGAGCAGTTAGTATTCCTG), Mm GAPDH (F:TCAACAGCAACTCCCACTCCTCCA, R:ACCCTGTTGCTGTAGCCGGTATTCA), Mm β-ACTIN (F:GCAAGCAGGAGTACGATGA, R: CCATGCCAATGTTGTCTCTT).

### Western blotting

Cell lysates were resolved and transferred to PVDF membranes. Primary antibody against osteopontin (AF808, R&D systems) was used at 5 μg/ml, human p21 at 1:1000 (2947, Cell signalling) and β-actin (A5316, Sigma) at 1:5000. cPARP antibody was used at 1:1000 (9544, Cell signalling). Binding was detected with horse peroxidase-conjugated antibodies (7074 & 7076, Cell signalling, 1:5000) and images obtained with UVITEC Finealliance.

### Micocomputed tomography

Tibias were fixed in formalin and stored at 4 °C until needed. Bones were then mounted vertically in PBS and placed in the micro-CT scanner (SkyScan 1172, SkyScan) and scanned at an isotropic pixel size of 9 µm, 37 kV voltage, 228 mA current and using a 0.5 mm aluminium filter. Images were reconstituted and analysed using the SkyScan CT analyser software (Bruker) to quantify cortical bone lesions.

### Immunohistochemistry

OPN was detected in long bones using mouse OPN primary antibody at 15 μg/ml (AF808, R&D systems). Secondary rabbit antibody against goat was used at 1:200 dilution (P0160, Dako). Peroxidase reaction was performed using RTU Vectastin kit (PK7100, Vector) and impact DAB peroxidase substrate kit (SK4105, Vector). Then slides were counterstained with hematoxylin and mounted with DPX.

### Statistical analysis

Statistical analysis was performed using unpaired, two-tailed Student's *t*-test for comparisons between two groups and one-way ANOVA for comparison of more than two groups. For all experiments differences were considered significant at *P < 0.05, **P < 0.01 and ***P < 0.001. Results are presented as mean ±  SEM.

## Results

### Metformin has direct effects on preosteoblasts to increase myeloma cell adhesion

**Fig. 1:**
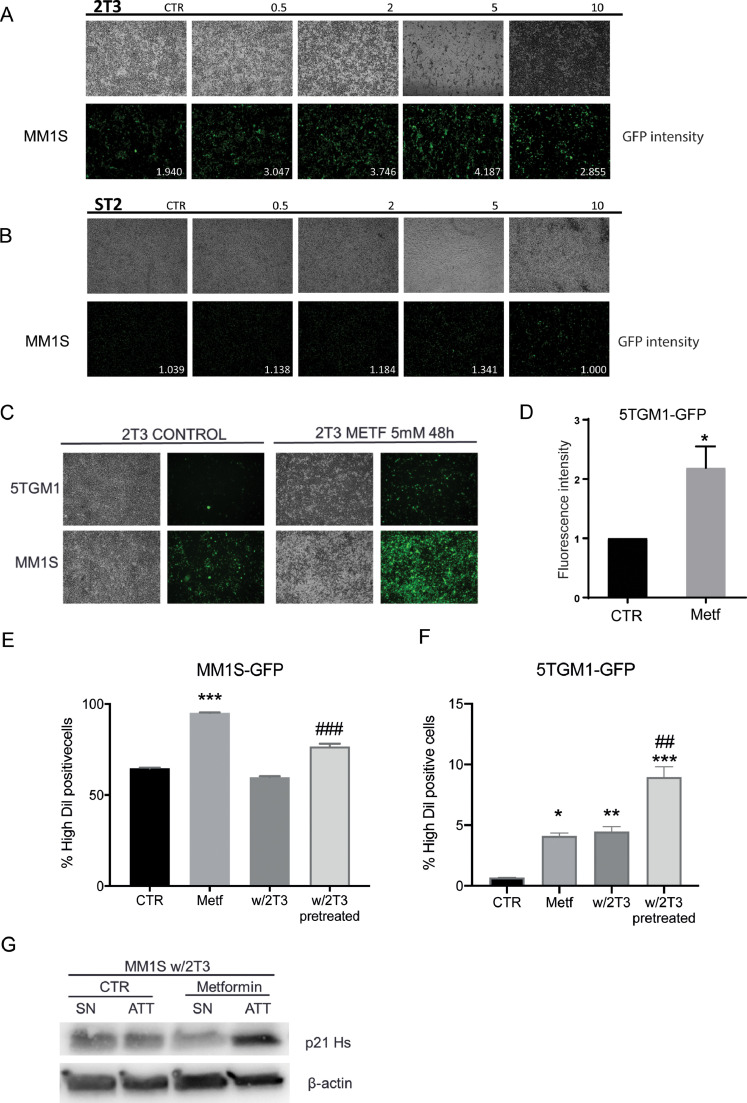
Metformin-pretreated 2T3 preosteoblasts increase myeloma cell adhesion and reduce proliferation in coculture. **(A)** 2T3 preosteoblasts and **(B)** ST2 stromal cells were treated with increasing doses of metformin (0.5–10 mM) for 48 h. Cells were then washed and MM.1S myeloma cells seeded on top. 72 h later supernatant was removed and cells imaged (4x magnification). GFP quantification is shown in each figure. **(C)** 5TGM1 or MM.1S myeloma cells were cocultured with metformin-pretreated 2T3 preosteoblasts (5 mM) as described previously. Images were visualised either by brightfield or fluorescence microscopy (4x magnification). **(D)** Quantification of fluorescence intensity of 5TGM1 cells attached to 2T3 after 3 days of coculture (*n* = 5). *p < 0.05. Metf = metformin **(E)** MM.1S myeloma cells were cultured in the presence and absence of 2T3 preosteoblasts that had been pretreated or not with metformin. Data are expressed as the proportion of Dil high positive cells at day 4. *** *p* < 0.001 as compared to control, ### *p* < 0.001 as compared to myeloma cells cultured with 2T3. Representative graph of 6 independent experiments. **(F)** 5TGM1-GFP cells were cultured in the presence of 2T3 preosteoblasts that had been pretreated or not with metformin. Data are expressed as the proportion of high violet staining, * *p* < 0.05, ** *p* < 0.01 and ***p < 0.001 as compared to control. ## *p* < 0.01 as compared to myeloma cells cultured with 2T3. Representative graph of 3 independent experiments. (**G)** 2T3 cells were treated with metformin for 48 h, washed with media and then MM.1S-GFP myeloma cells were seeded on top and cocultured for 72 h. Supernatant (SN) and attached (ATT) myeloma cells were removed from the coculture and p21 expression determined. All data are presented as mean ± SEM.

To investigate the effects of metformin on bone marrow microenvironment cells, 2T3 preosteoblasts and the bone marrow stromal cell line ST2 were used. We implemented an *in vitro* pretreatment coculture system where 2T3 or ST2 cells were treated for 48 h with metformin, followed by seeding of myeloma cells on preosteoblasts or stromal cells. To avoid a direct effect of metformin on myeloma cells, culture media was removed after metformin treatment and adherent cells were washed before the addition of myeloma cells. Treatment of 2T3 cells with increasing concentrations of metformin resulted in a dose-dependent increase in MM.1S-GFP myeloma cell adhesion, as demonstrated by an increase in GFP signal (Fig. 1A). In contrast, pretreatment of bone marrow stromal cells with metformin had no effect on myeloma cell adhesion, suggesting that the effect of metformin to promote myeloma cell adhesion may be specific to preosteoblasts (Fig. 1B). An increase in myeloma cell adhesion was detected after only 6 h of coculture (Fig. S1). Metformin had a limited effect on 2T3 viability, with a small reduction in viability following treatment with 5 mM–20 mM metformin (Fig. S2), likely responsible for the decrease in adherence observed with 10 mM metformin. Treatment with metformin, either continuously or a 48 h pulse, was also found to increase the osteogenic capacity of 2T3 preosteoblasts (Fig. S3). As with MM.1S cells, metformin pretreatment of 2T3 preosteoblasts was found to increase adhesion of 5TGM1 myeloma cells (Fig. 1C&D). Similarly, metformin pretreatment of MG63 osteoblast-like cells was found to increase adhesion of MM.1S myeloma cells (Fig. S4).

To further study the indirect effects of metformin-treated preosteoblasts on myeloma cell proliferation, we utilised membrane proliferation markers where the fluorescent intensity of the dye is lost during cell division (Fig. S5). Using this approach, we tracked changes in myeloma cell division in cocultures of myeloma cells and preosteoblasts where preosteoblasts had been previously exposed to metformin. Coculture of myeloma cells with 2T3 cells had no significant effect on myeloma cell proliferation. Direct treatment of either MM.1S-GFP or 5TGM1-GFP myeloma cells with metformin resulted in a significant increase in DiL (violet high) positive cells, indicative of a reduction in proliferation (Fig. 1E&F) and confirming previous reports that metformin can act directly on myeloma cells to reduce proliferation. Notably, when 2T3 preosteoblasts were pretreated with metformin, a significant increase in DiL/violet-positive, non-dividing myeloma cells was detected after 4 days of coculture (Fig. 1E&F). To better understand the nature of the reduction in proliferation, we examined the expression of p21(p21^Cip1^), a protein involved in cell cycle progression that acts as a repressor of cell cycle. Using the same coculture model with metformin-pretreatment of preosteoblasts, an increase in p21 expression was observed in MM.1S cells attached to metformin-pretreated preosteoblasts (Fig. 1G). To study the direct effect of metformin on myeloma cells, we used a panel of myeloma cell lines to confirm a dose-dependent direct induction of apoptosis by metformin. Higher doses of metformin were required for the direct apoptotic effect in myeloma cells compared to doses required for indirect effects via osteoblasts. Metformin-induced apoptosis was reduced by contact with ST2 bone marrow stromal cells, supporting a role for the microenvironment in the response to metformin (Fig. S6). Together, our results suggest that metformin can have specific indirect effects via preosteoblasts to increase myeloma cell adhesion and reduce cell division indicative of a reduction in proliferation or increase in quiescence.

### Myeloma cell adhesion to metformin-pretreated preosteoblasts is mediated through osteopontin

**Fig. 2:**
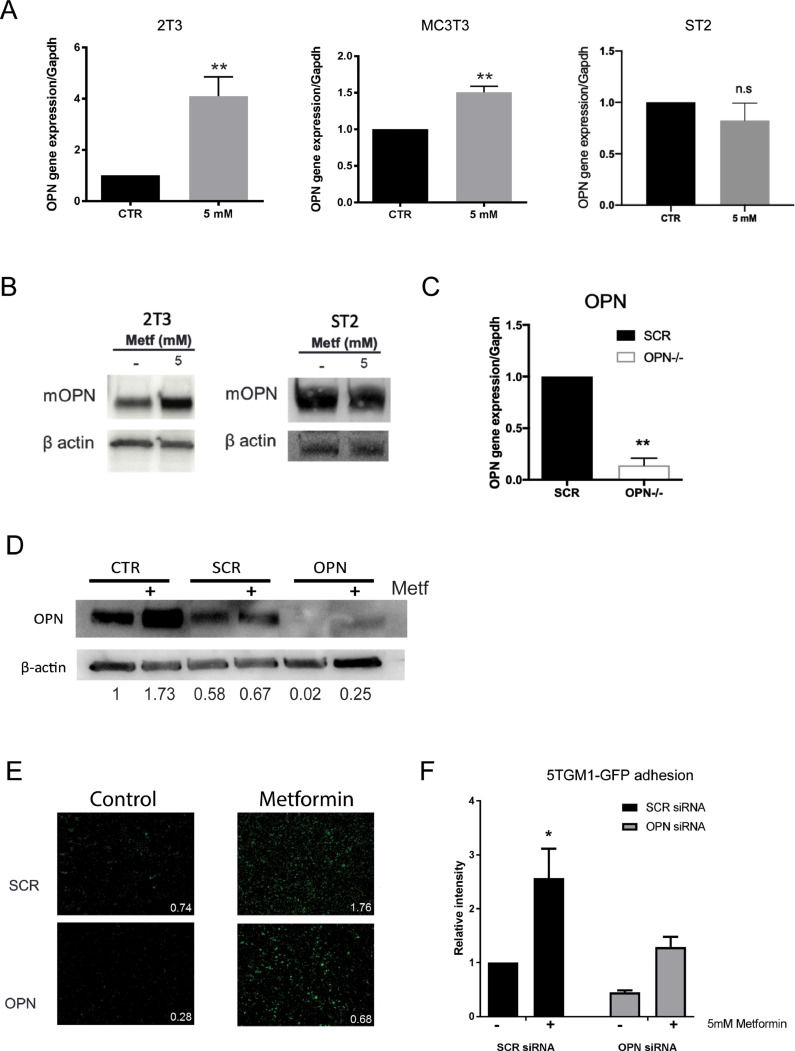
Metformin treatment increases OPN expression in preosteoblasts, contributing to an increase in adhesion of myeloma cells. **(A)** 2T3, MC3T3 and ST2 were treated with 5 mM metformin for 48 h and osteopontin gene expression quantified (** *p* < 0.01 as compared to control, *n* = 3–7). **(B)** Osteopontin protein expression following metformin treatment for 48 h in 2T3 preosteoblasts and ST2 stromal cells. **(C)** OPN gene expression in 2T3 preosteoblasts after transfection with either scrambled (SCR) or OPN siRNA (** *p* < 0.01 as compared to scrambled transfection, *n* = 3). **(D)** OPN protein expression in 2T3 preosteoblasts following transfection with OPN siRNA or scrambled control SCR and treatment with metformin. CTR = untransfected. 2T3 preosteoblasts (scrambled control (SCR) or osteopontin siRNA (OPN)) were pretreated with metformin and adhesion imaged and quantified. (**E)** Representative image and quantification of specific image. **(F)** Quantification of myeloma cell adhesion (* *p* < 0.05 as compared to control, *n* = 4). Data are presented as mean ± SEM.

We have shown that metformin has a direct effect on preosteoblasts that increases the subsequent adhesion of myeloma cells. Adhesion of myeloma cells to osteoblasts within the endosteal niche is known to be a pivotal mechanism to support myeloma localisation. Osteopontin (OPN) is reported to positively regulate solid tumour cell proliferation and metastasis and has been recently implicated in tumour dormancy [40]. Since OPN is a chemoattractant expressed by osteoblasts and well known to play a role in bone cell homing, including multiple myeloma [40,41], we sought to investigate whether the effect of metformin to support myeloma cell adhesion and promote myeloma development was mediated via OPN. Treatment of 2T3 and MC3T3 preosteoblasts with metformin revealed a significant increase in OPN gene and protein expression (Figs. 2A&B, S7). In contrast, treatment of ST2 bone marrow stromal cells with metformin had no effect on osteopontin gene or protein expression (Fig. 2A&B). To further study the role of osteoblast-derived OPN in myeloma cell adhesion, loss-of-function experiments were performed using small interference RNA strategies. OPN specific siRNAs were used to successfully reduce OPN gene expression in 2T3 preosteoblasts (Figs. 2C, S8) and to reduce OPN protein in response to treatment with metformin when compared to scrambled control (Fig. 2D). Genetic knockdown of osteopontin expression in preosteoblasts was found to reduce the increase in myeloma cell adhesion in response to metformin pretreatment of 2T3 preosteoblasts (Fig. 2E&F). Taken together, these results indicate that osteoblast-derived OPN is at least partially responsible for the indirect effects of metformin to promote myeloma cell attachment to preosteoblasts.

### Metformin pretreatment increases myeloma tumour burden and bone disease *in vivo*

**Fig. 3:**
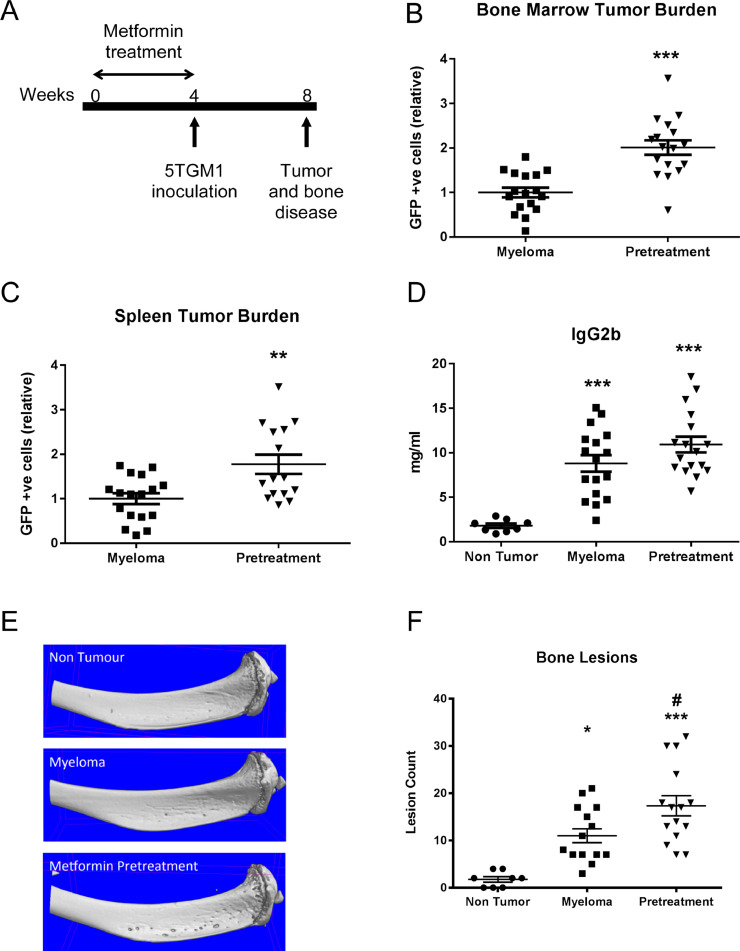
Pretreatment with metformin increases myeloma tumour burden and osteolytic bone lesions. **(A)** C57Bl/KaLwRij mice were treated with 2.5.mg/ml metformin or vehicle control for four weeks prior to cessation of treatment and inoculation of 1 × 10^6^ 5TGM1 myeloma cells. (Control *n* = 8, myeloma, *n* = 17, metformin *n* = 17). The proportion of GFP-positive 5TGM1 myeloma cells in bone marrow **(B)** and spleen **(C)** was quantitated by flow cytometry (** *p* < 0.01, *** *p* < 0.001 as compared to myeloma control). **(D)** Serum paraprotein was quantitated by IgG2b ELISA (*** *p* < 0.001 as compared to non-tumour bearing mice). **(E)** Representative images of osteolytic bone lesions. **(F)** MicroCT analysis and quantification of osteolytic bone lesions (*p < 0.05, ***p < 0.001 as compared to non-tumour bearing mice; # *p* < 0.05 as compared to myeloma-bearing control. Data are presented as mean ± SEM.

Our *in vitro* studies suggest that metformin has an indirect effect on myeloma cells to increase myeloma cell adhesion and quiescence, mediated at least in part through preosteoblast-derived OPN. To determine whether this indirect effect can be observed *in vivo*, we combined the well-characterised 5TGM1 murine model of myeloma with a metformin pre-treatment strategy, allowing us to exclude direct effects of metformin on myeloma cells. C57BL/KaLwRij mice were treated with metformin for 4 weeks at which point treatment was halted, prior to inoculation of 5TGM1-GFP myeloma cells (Fig. 3A). C57BL/KaLwRij mice were treated with 2.5 mg/ml metformin in drinking water for 4 weeks, a dose previously shown to be clinically relevant. Metformin was well tolerated, with no detectable adverse effects, changes in body weight or water consumption and no changes in glucose levels (Fig. S9). Metformin pretreatment had no effect on either circulating levels of RANKL and OPG or local concentrations in the bone marrow plasma at 4 weeks (Fig. S10).

**Fig. 4:**
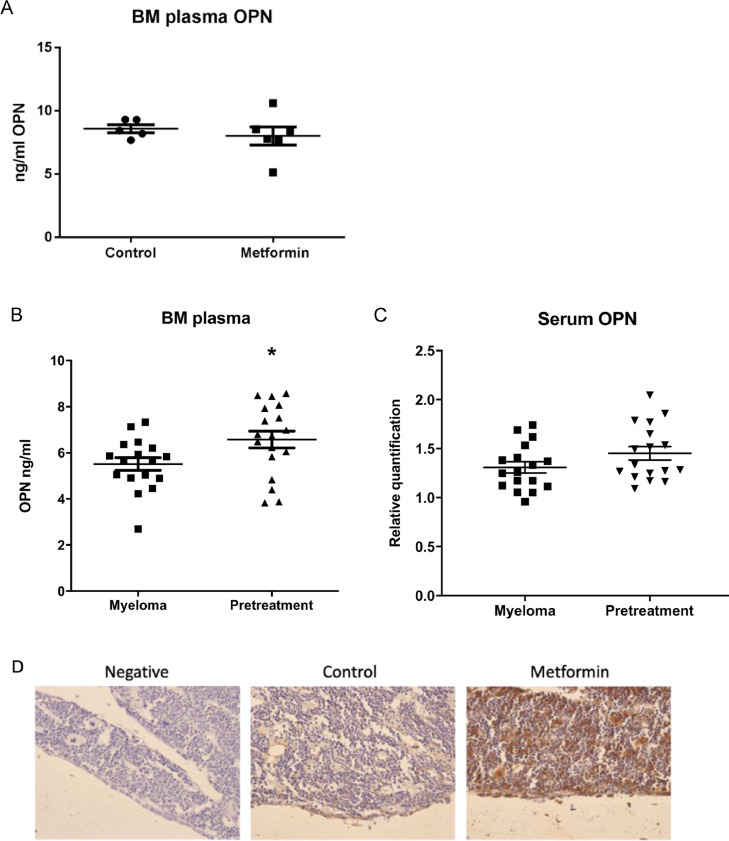
Metformin pretreatment *in vivo* increases OPN levels in the bone microenvironment. **(A)** Osteopontin quantification in the bone marrow plasma of C57Bl/KaLwRij mice treated for 4 weeks with metformin (males and females were used, control *n* = 5, metformin *n* = 6). Osteopontin levels in bone marrow plasma **(B)** or serum **(C)** of myeloma-bearing mice pretreated with vehicle control (myeloma) or metformin (pretreatment) (*p < 0.05, myeloma *n* = 17, metformin *n* = 17). **(D)** OPN immunohistochemistry showing OPN expression in the bone marrow of metformin pretreated mice compared to control. Magnification 40x.

Pretreatment with metformin resulted in a significant increase in tumour burden, reflected by a doubling of the proportion of myeloma cells within the bone marrow and spleen, and elevated serum paraprotein (Figs. 3B, C&D, S11, S12). Myeloma-bearing mice developed a characteristic osteolytic bone disease, with microCT analysis demonstrating significantly more osteolytic lesions in mice pretreated with metformin (Fig. 3E&F). While we found no increase in OPN in the bone marrow plasma of mice treated with metformin for 4 weeks (Fig. 4A), metformin pretreatment elevated OPN levels in the bone marrow plasma, but not serum, of myeloma-bearing mice (Fig. 4B&C). Immunohistochemistry provided further evidence for an increase in osteopontin in the bone marrow of mice pre-treated with metformin (Fig. 4D).

## Discussion

In recent years there has been widespread interest in investigating metformin as an anti-tumour and anti-ageing therapeutic. While preclinical studies support a direct anti-tumour effect of myeloma, to date the contribution of the bone microenvironment has been largely ignored. Given the interdependence of myeloma cells with the bone microenvironment, particularly with bone-lining osteoblasts, and the conflicting reports of metformin in bone biology, we aimed to investigate whether metformin could have an indirect effect in myeloma via bone cells. In the current work we show how metformin acts upon preosteoblasts to increase the subsequent adhesion of myeloma cells, with this indirect effect on myeloma cells dependent, at least in part, upon elevated OPN levels in metformin-treated preosteoblasts. *In vivo*, we implemented a pretreatment model where mice were treated with metformin prior to tumour inoculation, allowing us to eliminate direct anti-tumour effects. Unexpectedly, metformin pretreatment was found to increase myeloma tumour burden and myeloma bone disease, with metformin treatment associated with an increase in osteopontin within the bone microenvironment. Altogether, our results show that metformin treatment increases OPN expression and osteogenic potential in preosteoblasts, increasing their capacity to harbour myeloma cells and potentially expanding the endosteal niche. *In vivo*, this may translate to myeloma cells adhering more efficiently to these niches and thus, worsening the disease outcome.

Osteoblasts play a critical role in the homing and maintenance of cancer cells within the bone marrow. It is known that osteoblasts contribute to several cancers by creating a favourable environment for the cells to home to the bone and metastasize [42]. In the same way, they contribute to myeloma by supporting myeloma cell growth and survival. This happens in part by the ability of osteoblasts to secrete IL-6 when they are in coculture with myeloma cells [43]. Osteoblasts are also involved in cell-to-cell interactions which facilitate homing of myeloma circulating cells [44]. Thus, the bone niche has been acknowledged as the site for myeloma cells to home and initially reside within the bone marrow and it has opened a large area of research to further study and understand the disease [36]. The bone marrow niche plays a vital role in homing, differentiation and proliferation of malignant plasma cells, with several studies highlighting the importance of osteoblasts in myeloma cell quiescence and dormancy [36,37]. We provide evidence to suggest that metformin impacts the bone marrow niche with a significant increase in myeloma cell adhesion to preosteoblasts in response to prior treatment of osteoblasts with metformin. Interestingly, the bone marrow stromal cell line ST2 did not support changes in myeloma cell adhesion highlighting the specific effect of metformin on osteoblasts. Our studies elucidate changes in myeloma cell proliferation in response to metformin effects on preosteoblasts. The use of proliferation markers allowed us to explore the level of division of myeloma cells in coculture with pretreated osteoblasts. These results showed that myeloma cells presented a reduction in their proliferation rate together with an increase in p21 expression. Thus, the direct effects of metformin on preosteoblasts result in indirect effects on myeloma cells, including an increase in attachment.

Given our findings, and the reported osteogenic effects of metformin, it could be postulated that metformin may have a role in expanding the endosteal niche where dormant and/or quiescent myeloma cells reside. It is known that after tumour inoculation into C57Bl/KaLwRij mice, only a few colonizing myeloma cells will adhere to endosteal bone whereas the majority of myeloma cells will continue circulating through the bone marrow [37]. Thus, we speculated whether metformin, by the changes observed on osteoblasts *in vitro*, was able to create a more favourable bone marrow environment for the initial retention and subsequent expansion of tumour cells. To determine whether an indirect effect of metformin could impact the development of myeloma, we used the 5TGM1 mice model. C57/KaLwRij mice were treated with metformin for 4 weeks, with subsequent inoculation of myeloma cells following cessation of metformin treatment, to eliminate direct antitumour effects of metformin. The dose of metformin was previously shown to be clinically relevant [45,46] and was well tolerated. No effect of metformin on blood glucose levels was observed, which may reflect that the animals were not diabetic, or on a high-fat diet, and were not fasted prior to blood glucose analysis. Interestingly, pretreatment with metformin resulted in an increase in tumour burden and osteolytic bone lesions, suggesting that the effects of metformin on the host microenvironment indirectly promote myeloma. It is intriguing to consider and warrants further investigation as to whether the increased tumour burden *in vivo* may be indicative of an *in vivo* influence to activate quiescent myeloma cells that is not present in our *in vitro* culture systems, where metformin pretreatment increases adhesion and reduces proliferation, or whether it is a result of increased homing and/or adhesion of myeloma cells to bone. Notably, we observe an increase in tumour burden in both bone marrow and spleen, reflecting the haematopoietc nature of both sites in mice, but also suggesting the contribution of a systemic effect of metformin to promote myeloma cell homing. Current clinical evidence to support metformin use in MGUS and myeloma comes from diabetic patients, and diabetes has been associated with poor clinical outcomes in myeloma [10]. We acknowledge that our findings are limited to a non-diabetic myeloma model, and as such, do not interrogate the relationship between diabetes and myeloma development. While there are limited reports of such tumour-enhancing effects of metformin, the antioxidant antidiabetic agents saxagliptin, sitagliptin and α-lipoic acid have also been shown to accelerate metastasis in murine experimental metastasis models [47].

OPN is known to positively regulate solid tumour cell proliferation and metastasis and is a chemoattractant well known to play a role in bone cell homing, including multiple myeloma [40,41,48,49]. Metformin was found to increase osteopontin expression in a panel of osteoblasts and to induce cell attachment of different myeloma cell lines. Moreover, the genetic knockdown of osteopontin expression in osteoblasts resulted in a reduction of cell adhesion. *In vivo*, pretreatment with metformin significantly elevated OPN within the bone marrow microenvironment of myeloma-bearing mice, as measured by ELISA. Immunohistochemistry provided further support, with a clear increase in OPN expression in the bone marrow of non-tumour-bearing mice treated with metformin. In addition to cells of the bone microenvironment, myeloma cells are also known to express OPN [50,51], and it cannot be excluded that the increase in OPN reflects the elevated tumour burden observed following metformin pre-treatment. Notably, no such increase was observed in circulating OPN, suggesting that metformin has specific effects to elevate OPN *in vivo* within the myeloma-bone microenvironment. While a limitation of our study is the use of murine systems, the high homology between murine and human osteopontin supports the clinical relevance. While our data support an effect of metformin mediated at least in part via osteopontin, we cannot exclude the involvement of other adhesion molecules or chemokines such as N-cadherin, VCAM-1 or CXCL12.

Taken together, our results reveal a series of direct effects of metformin on the bone microenvironment that underly an unexpected indirect protumourigenic effect of metformin on myeloma progression. Metformin treatment of preosteoblasts results in increased myeloma cell attachment, due at least in part to elevated osteopontin expression. Our study demonstrates that pretreatment of myeloma-bearing mice with metformin has significant effects to worsen myeloma development, with increased tumour burden and bone disease. The interdependence between myeloma cells and cells of the bone microenvironment has long been known to support both tumour growth and bone disease. The importance of this cellular crosstalk is exemplified by our current study, where an unexpected *in vivo* drug response can be explained, at least in part, by indirect effects mediated by preosteoblasts and highlighting the importance of studying myeloma pathogenesis within a physiologically-relevant setting.

## CRediT authorship contribution statement

**Beatriz Gámez:** Conceptualization, Methodology, Formal analysis, Writing – original draft, Writing – review & editing. **Emma V. Morris:** Methodology. **Sam W.Z. Olechnowicz:** Methodology. **Siobhan Webb:** Conceptualization, Methodology. **James R. Edwards:** Methodology. **Aneka Sowman:** Methodology. **Christina J. Turner:** Methodology. **Claire M. Edwards:** Conceptualization, Writing – review & editing, Supervision, Project administration, Funding acquisition.

## Declaration of Competing Interest

The authors declare no conflict of interest.

## Appendix Supplementary materials

Image, application 1
