# Supplementary material for: The antidiabetic drug metformin acts on the bone microenvironment to promote myeloma cell adhesion to preosteoblasts and increase myeloma tumour burden in vivo
Source: Transl Oncol. 2021 Dec 8;15(1):101301. doi: 10.1016/j.tranon.2021.101301 (PMC8665410; doi:10.1016/j.tranon.2021.101301)
Supplement: Supplementary file 1 [file mmc1.zip › mmc1.pdf]

SUPPLEMENTARY INFORMATION

The antidiabetic drug metformin acts on the bone microenvironment to promote myeloma cell adhesion to preosteoblasts and increase myeloma tumour burden in vivo

Beatriz Gámez[1,2], Emma V. Morris[1,2], Sam WZ. Olechnowicz[3], Siobhan Webb[1], James R. Edwards[3], Aneka Sowman[3], Christina J. Turner[1] and Claire M. Edwards[1,2,3*]

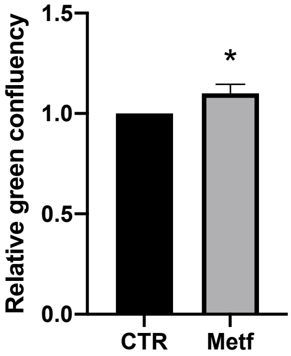

Supplementary Figure S1. Metformin treatment of 2T3 osteoblasts increases myeloma cell adhesion. 2T3 osteoblasts were treated with metformin (5mM) for 48h and then media was washed and 5TGM1-GFP cells were seeded on top for 6 hours. Supernatant was then removed and attached myeloma cells were imaged using Incucyte. Images were quantified for GFP confluency and compared to GFP confluency on non-treated 2T3 cells (n=3). * p< 0.05.

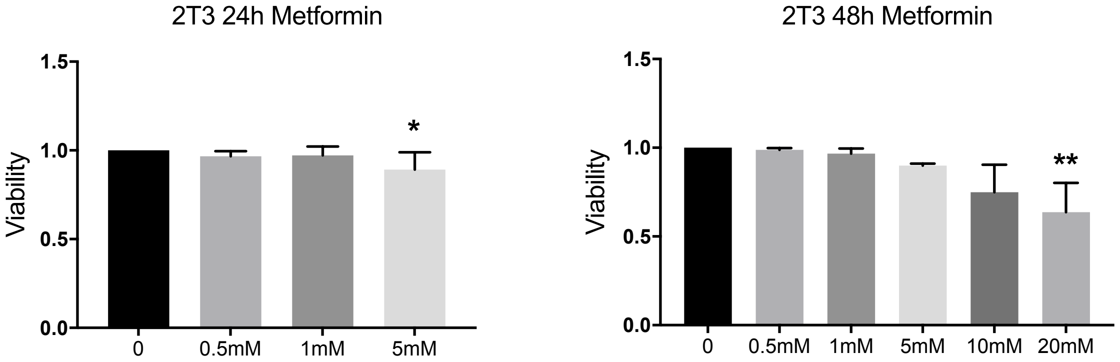

Supplementary Figure S2. Effect of metformin on 2T3 viability. 2T3 preosteoblasts were treated with a range of metformin doses for 24h or 48h and viability was analysed by Alamar Blue (n=4-9). *p<0.05, ** p<0.01 as compared to control.

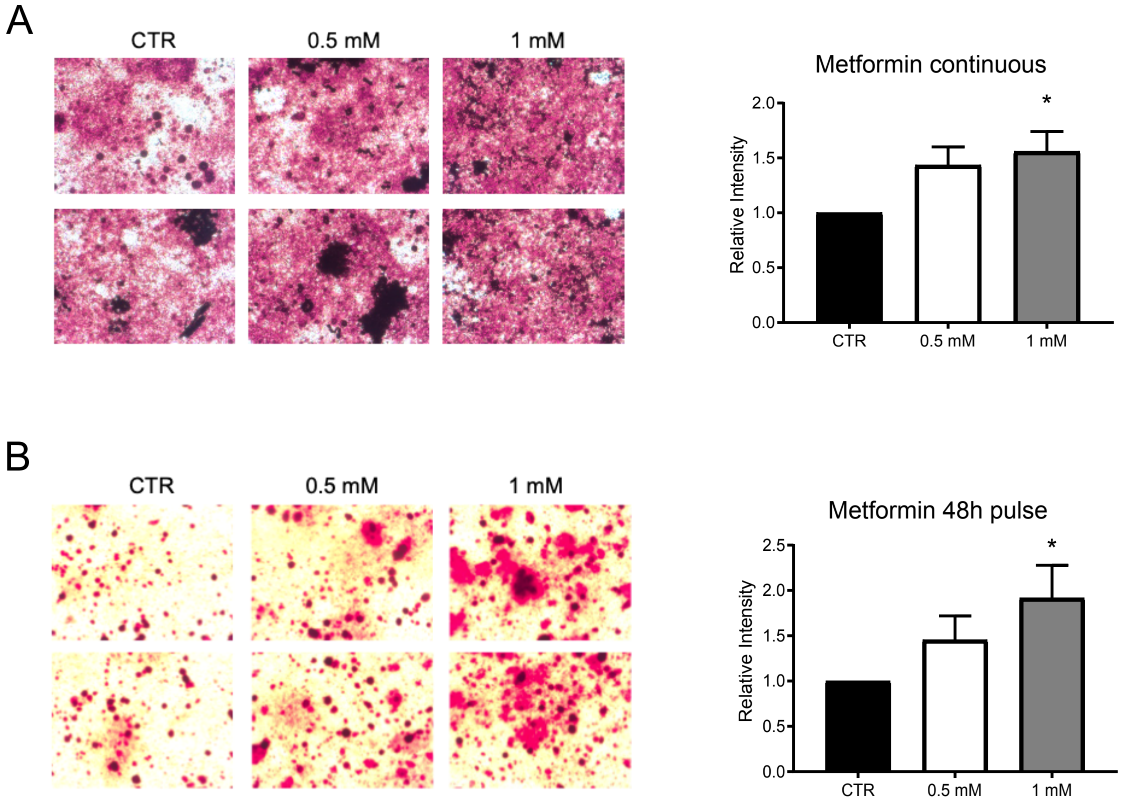

Supplementary Figure S3. Metformin increases osteogenic capacity. (A) 2T3 preosteoblasts were treated with metformin continuously during mineralisation for 20 days (n=5). (B) 2T3 preosteoblasts were treated with metformin for 48h and then mineralisation was performed under no metformin treatment (n=6-7). Cells were stained with alizarin red and the level of mineralisation was quantified using ImageJ (4x magnification). *p<0.05 as compared to control.

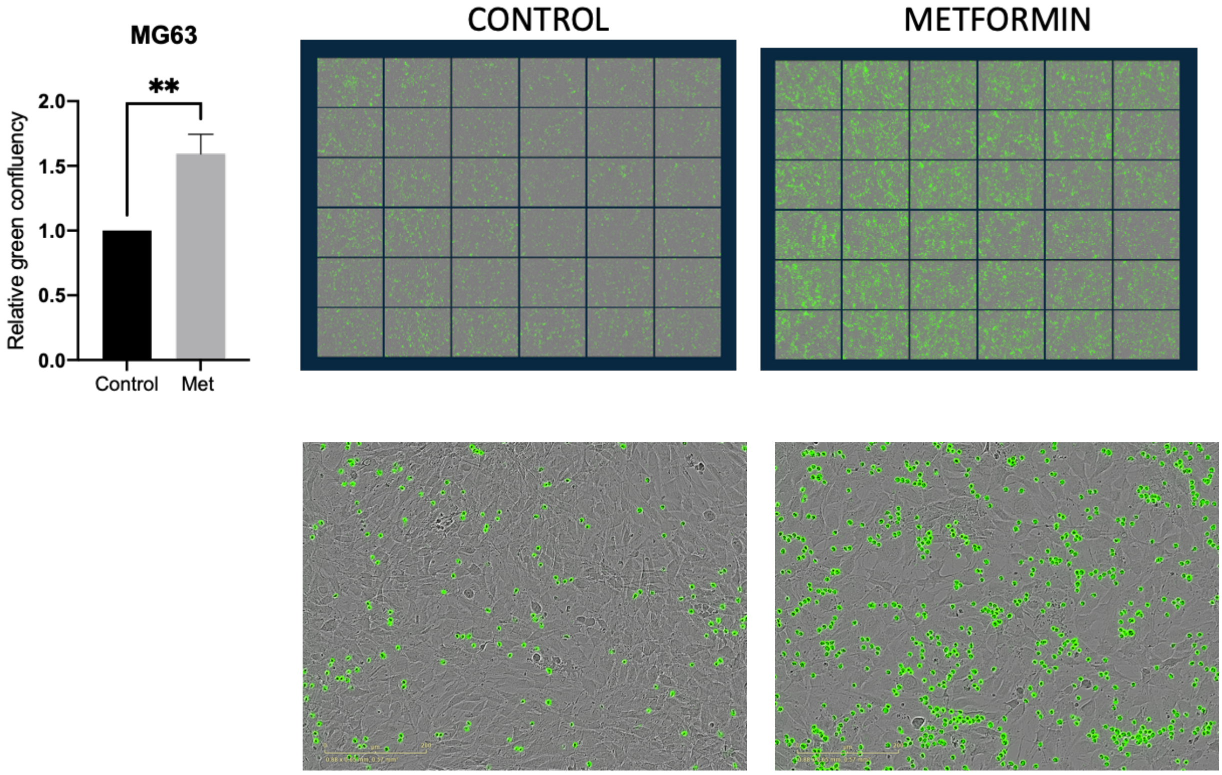

Supplementary Figure S4. Effect of metformin pretreatment of MG63 osteoblasts on myeloma cell adhesion. MG63 osteoblast-like cells were treated with metformin for 48h then MM1S-GFP myeloma cells were seeded on top for 72h (n=5). Incucyte images were taken at the end of the experiments when supernatant was removed. Relative green confluency was quantified using Incucyte software. Images on the top are an overview of all the images taken from a same well. Images on the bottom are a representation of a single image at 20x magnification used for quantification.. **p<0.01 as compared to control.

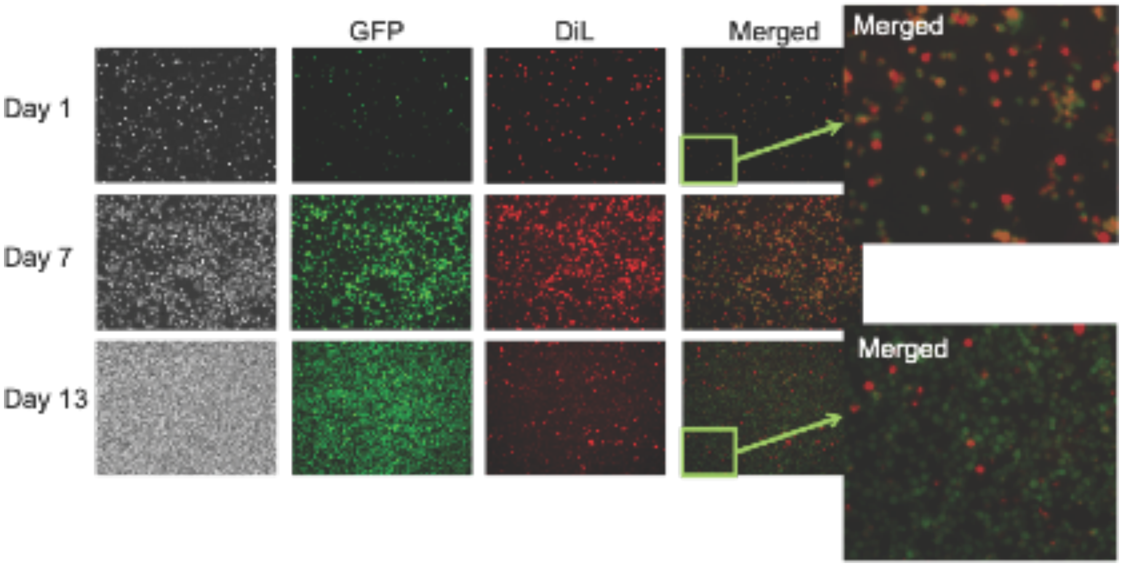

Supplementary Figure S5. Proliferation marker dilution over time. DiL proliferation marker was used to stain MM1S-GFP myeloma cells and track the proliferation of myeloma cells. Green indicates GFP positive myeloma cells. Red indicates uptake of DiL with no further cell division. Loss of red indicates cell division and loss of cell tracker dye. 10x magnification.

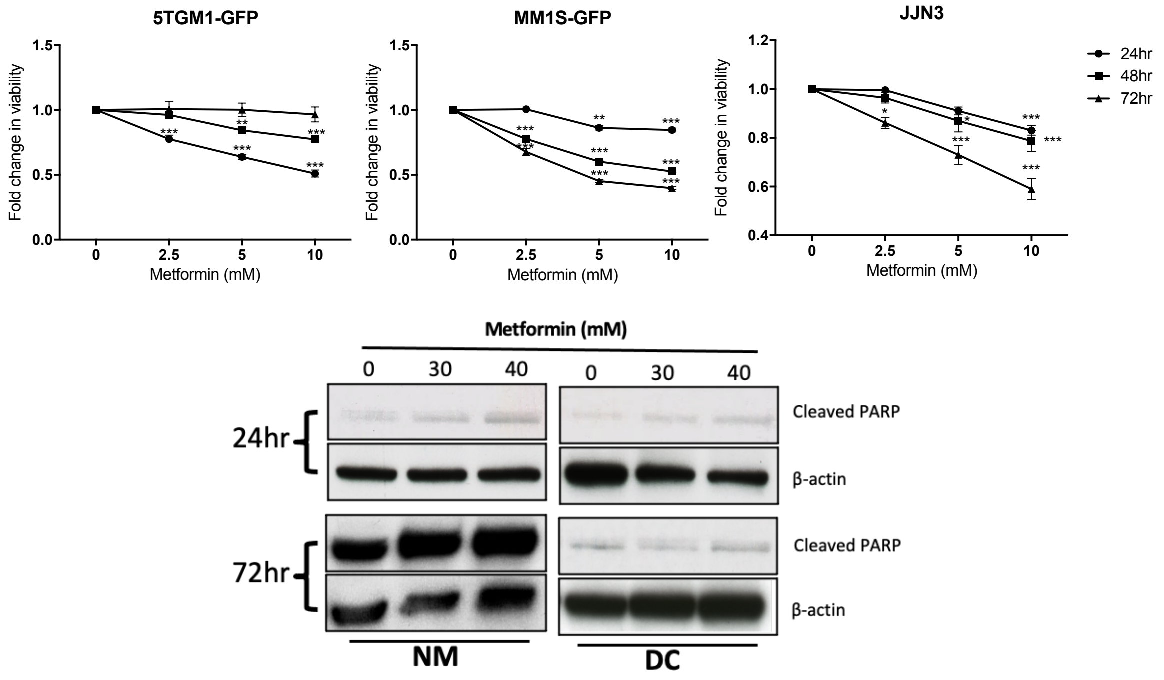

Supplementary Figure S6. Direct treatment of myeloma cells in single culture and coculture. (A) 5TGM1-GFP cells were treated with increasing doses of metformin for 24 - 72 hours. Viability was assessed by Alamar Blue and compared to time 0. (B) 5TGM1-GFP cells were cultured alone or in direct culture with HS5 stromal cells and levels of human cleaved PARP were detected by western blot.

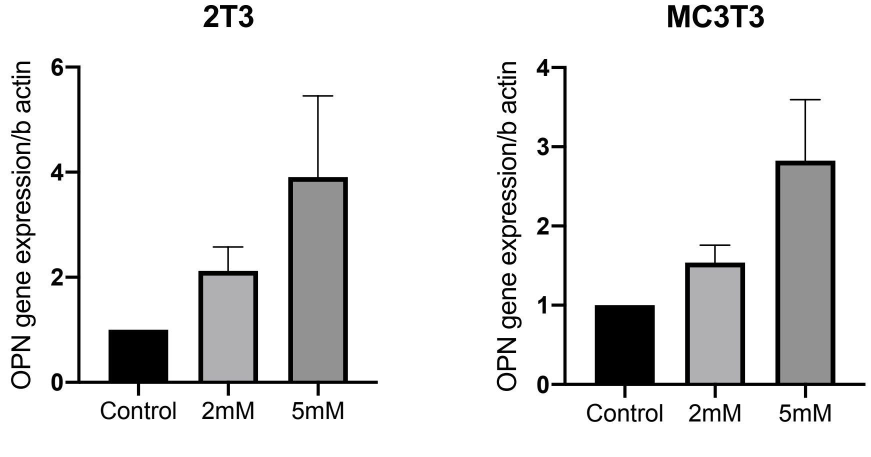

Supplementary Figure S7. Metformin treatment increases OPN expression in osteoblasts. Treatment with the indicated doses of metformin for 48h induced an increase in osteopontin gene expression in 2T3 and MC3T3 osteoblasts (n=3-4).

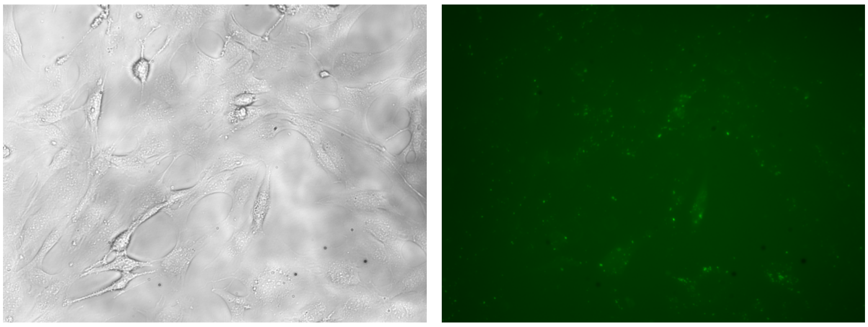

Supplementary Figure S8. Knockdown of osteopontin expression in 2T3 osteoblasts using siRNA. 2T3 osteoblasts were transfected with a FITC-tagged control siRNA and transfection confirmed by microscopy (left: bright field, right: fluorescence image, 10x magnification).

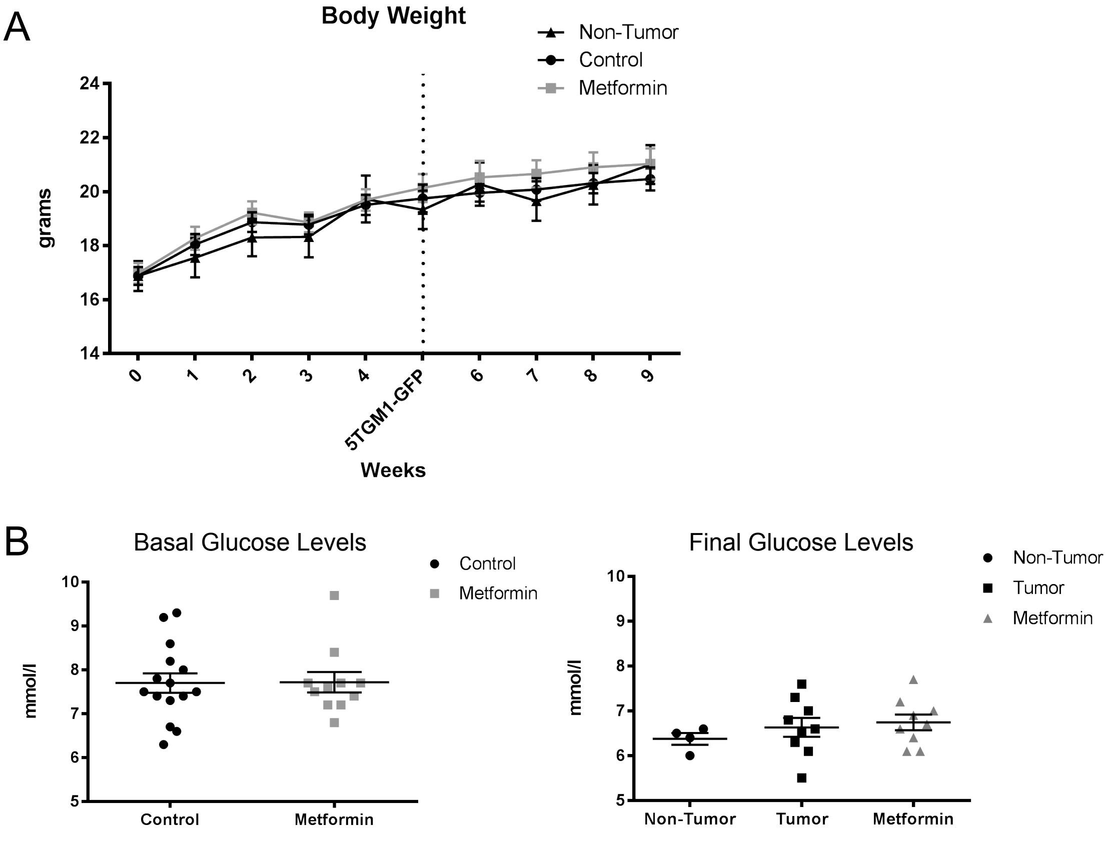

Supplementary Figure S9. Metformin has no effect on body weight or blood glucose. C57Bl/KaLwRij mice were treated with 2.5.mg/ml metformin or vehicle control for four weeks prior to cessation of treatment and inoculation of 1x10^6 5TGM1 myeloma cells. **(A)** Body weight over time (n=4-13). **(B)** Blood glucose prior to tumour inoculation (n=11-15) and prior to cull (n=4-9).

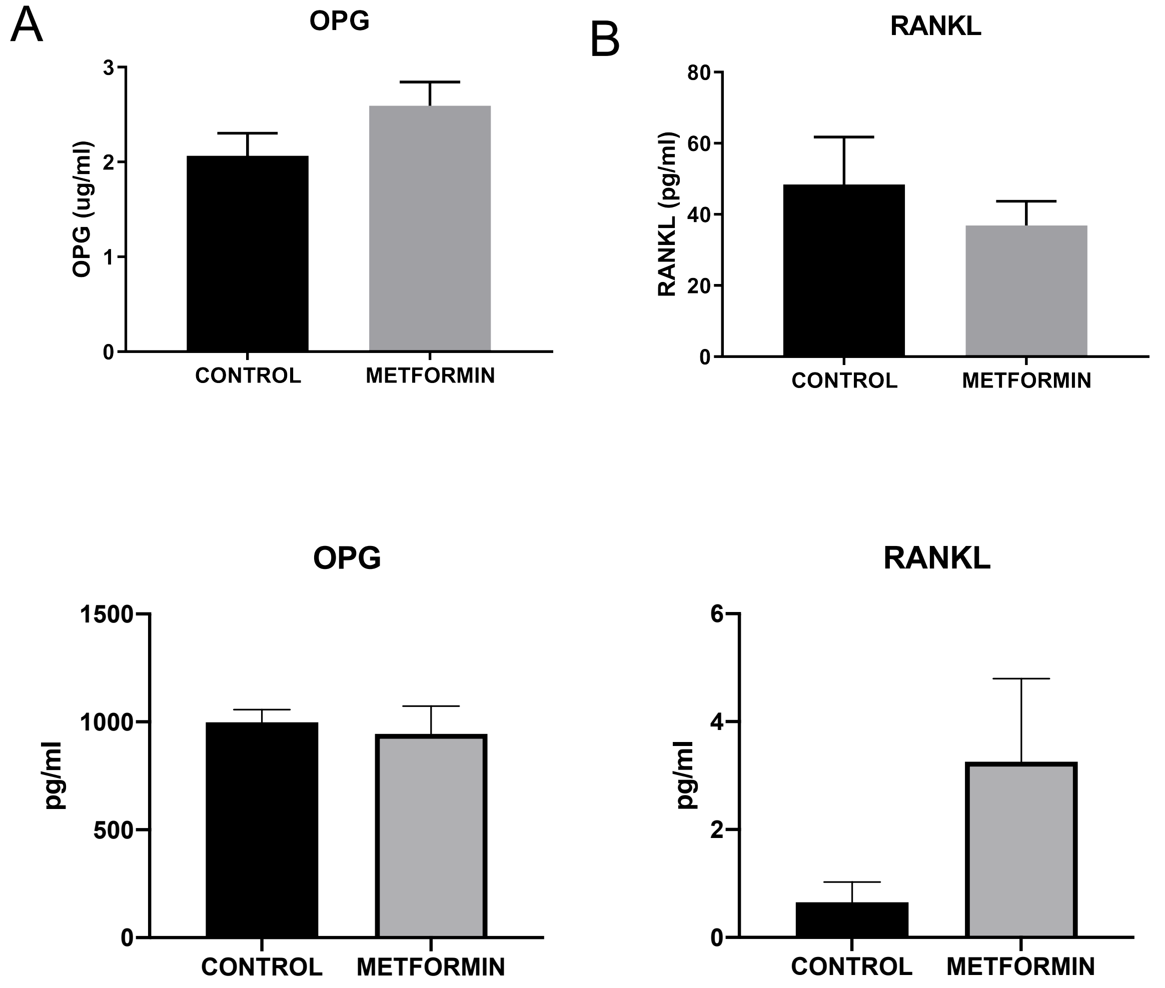

Supplementary Figure S10. Metformin has no effect on circulating or local levels of RANKL and OPG. C57Bl/KaLwRij mice were treated with metformin or vehicle control for four weeks. (A) OPG and (B) RANKL serum OPG (n=8-11) and RANKL (n=8-12). (C) OPG (n=5), (D) RANKL (n=5-6) in bone marrow plasma

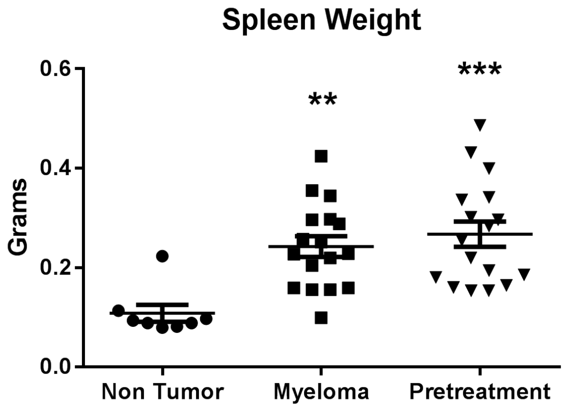

Supplementary Figure S11. Effect of metformin pretreatment on spleen weight in myeloma-bearing mice. C57Bl/KaLwRij mice were treated with 2.5.mg/ml metformin or vehicle control for four weeks prior to cessation of treatment and inoculation of 1x10^6 5TGM1 myeloma cells. Whole spleen weight is presented. ** p<0.01, *** p<0.001 as compared to non-tumour, n=8-17).

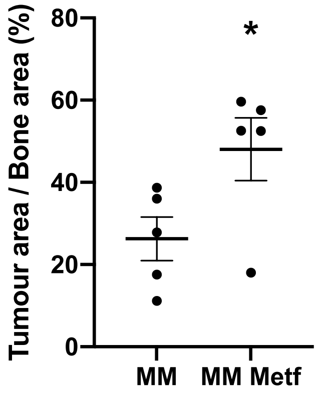

Supplementary Figure S12. Histomorphometric analysis of tumour area within bone. Metformin treatment was administered for 4 weeks and then myeloma 5TGM1-GFP cells were inoculated. Tumour areas were quantified and compared to total bone area (n=5).
