# Supplementary material for: The antidiabetic drug metformin acts on the bone microenvironment to promote myeloma cell adhesion to preosteoblasts and increase myeloma tumour burden in vivo
Source: Transl Oncol. 2021 Dec 8;15(1):101301. doi: 10.1016/j.tranon.2021.101301 (PMC8665410; doi:10.1016/j.tranon.2021.101301)

Supplementary Figure 6

5TGM1-GFP NM beta actin 24h

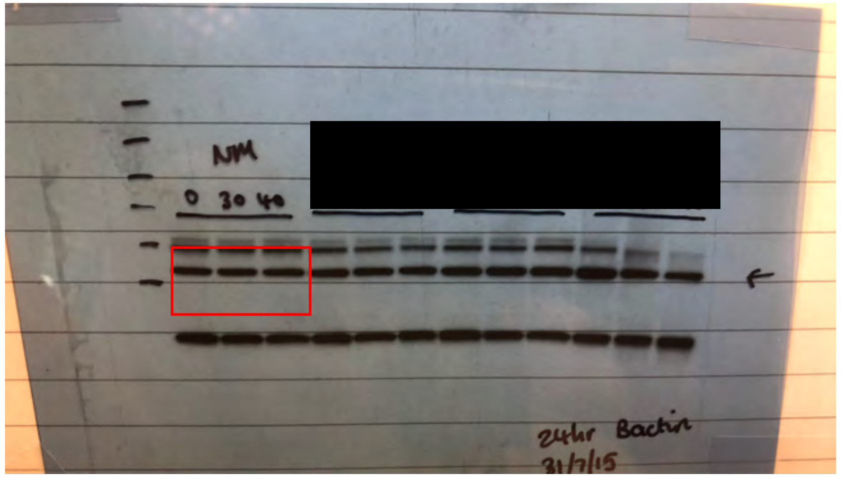

5TGM1-GFP NM cPARP 24h

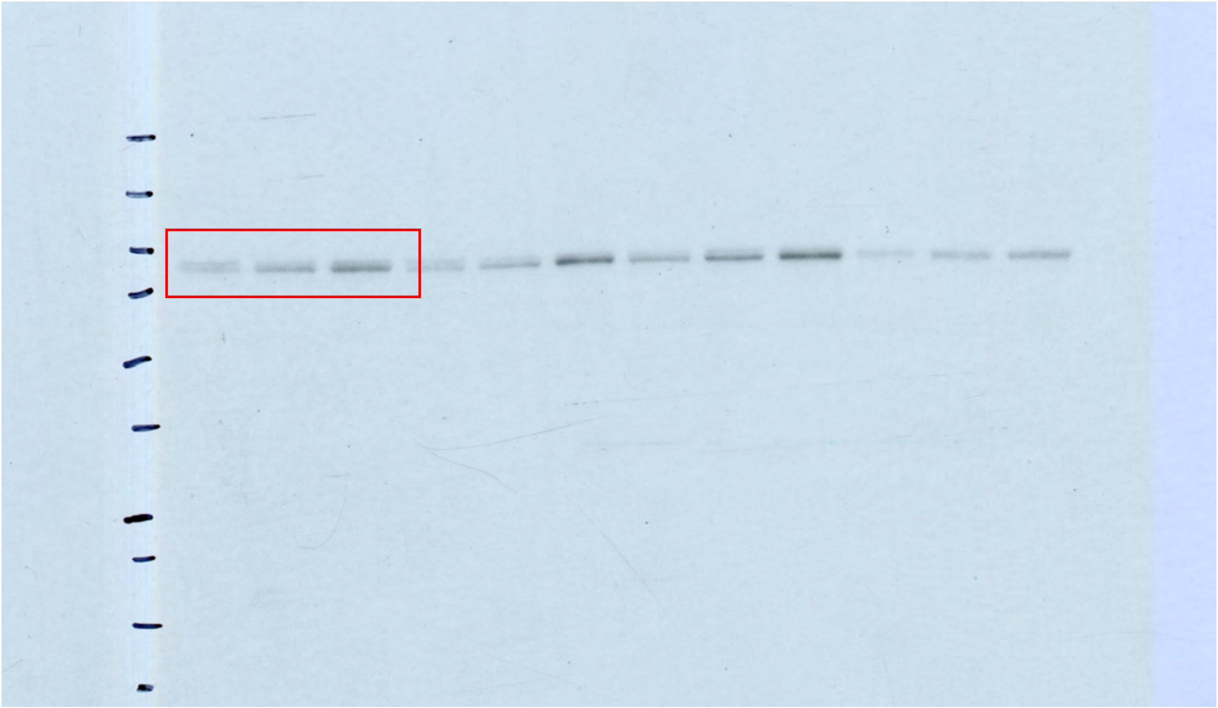

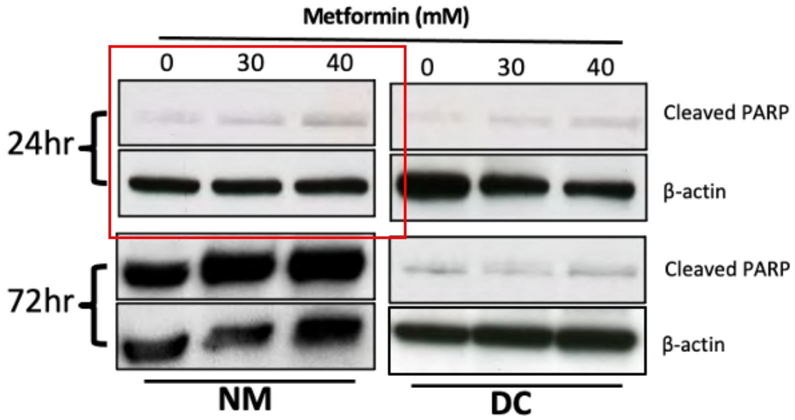

5TGM1-GFP NM beta actin 72h

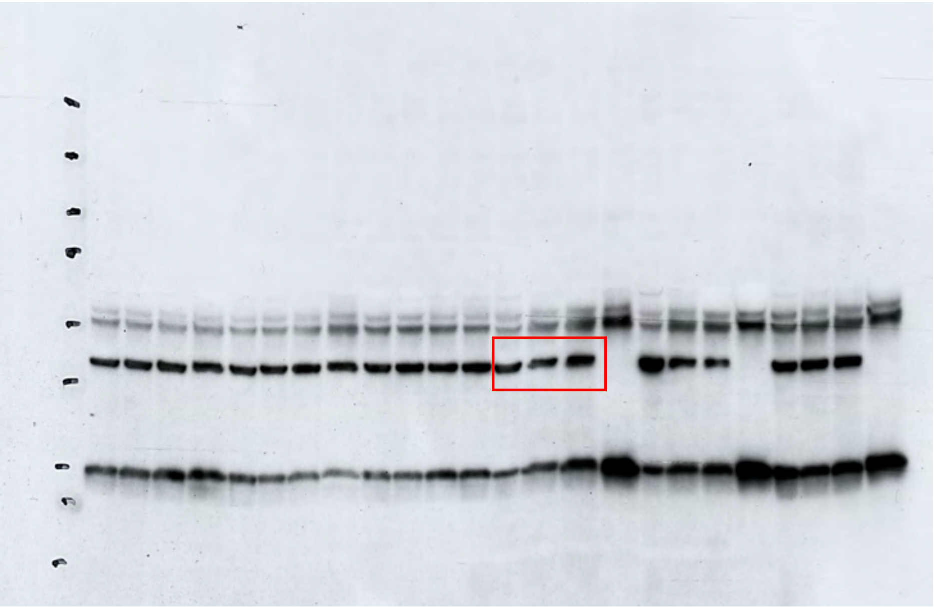

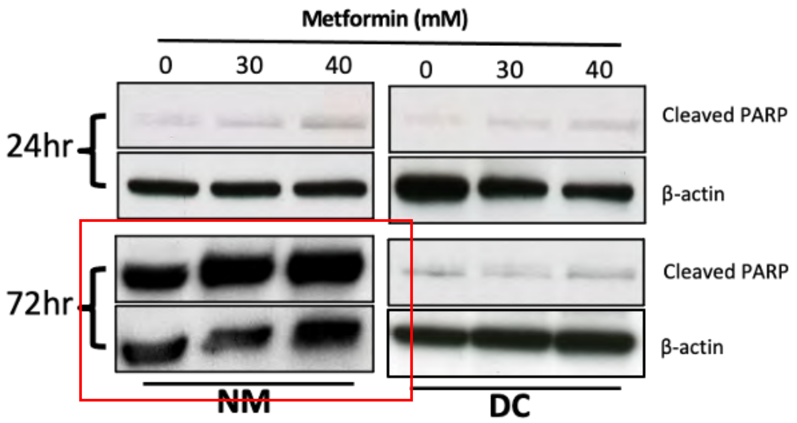

5TGM1-GFP NM cPARP 72h

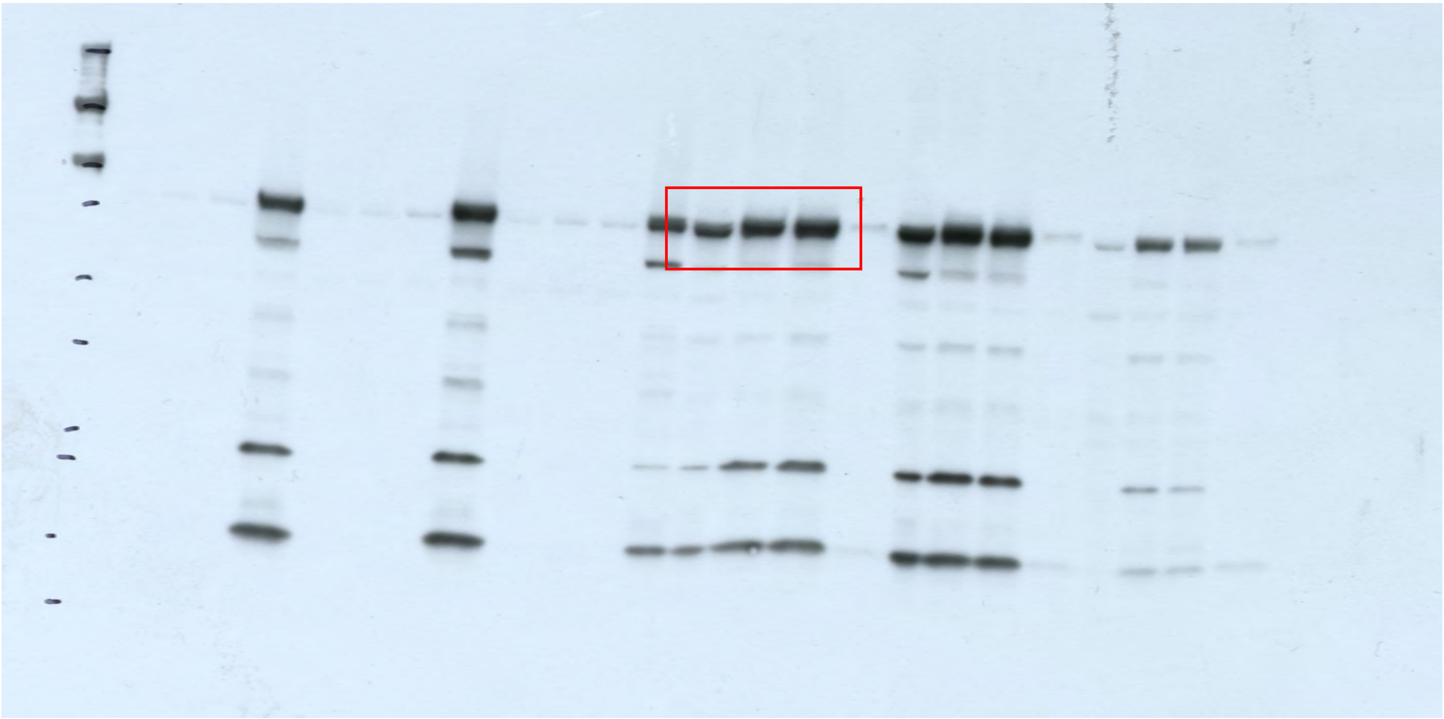

5TGM1-GFP CD c parp 72h

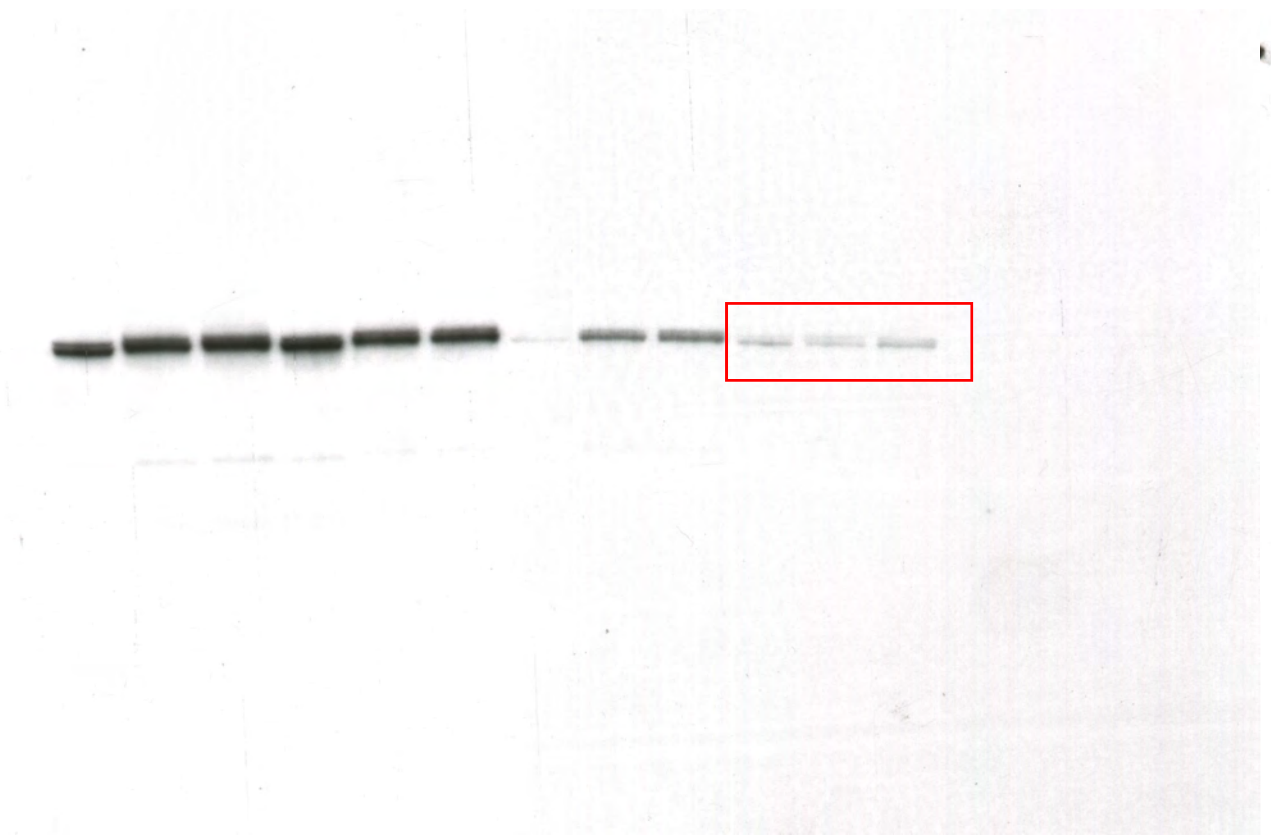

5TGM1-GFP CD b actin 72h

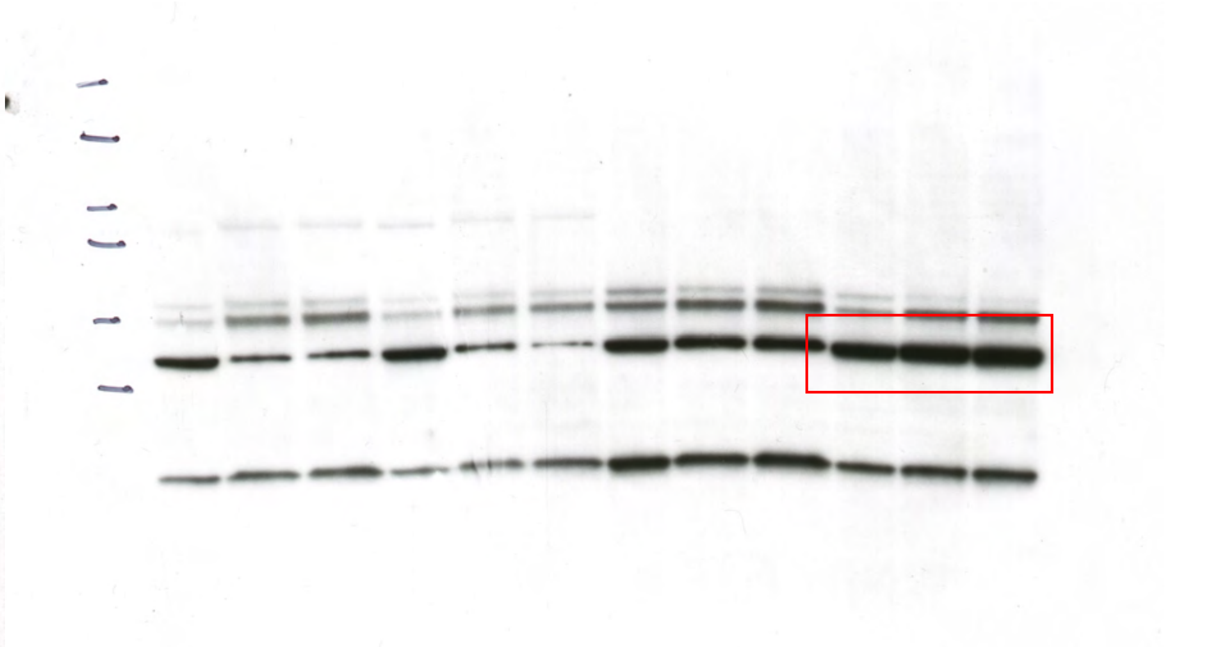

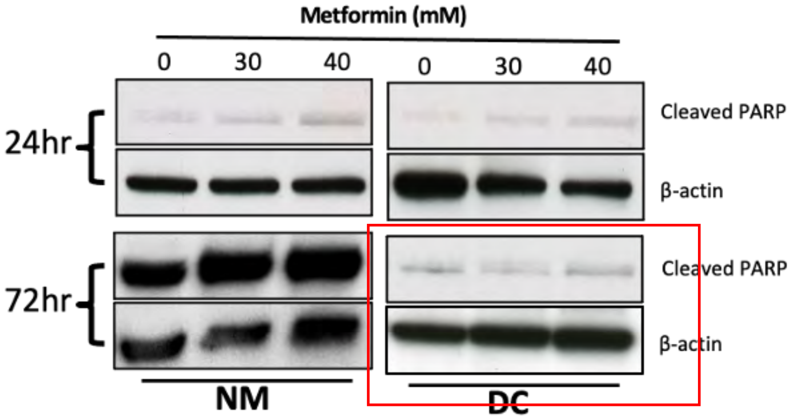

5TGM1-GFP DC beta actin 24h

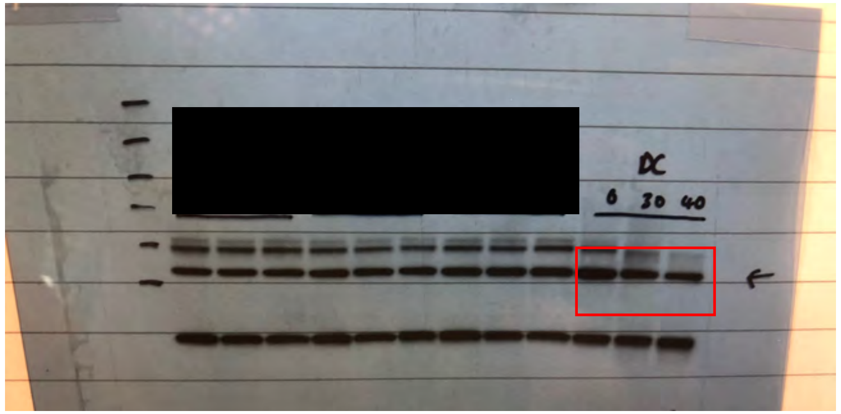

5TGM1-GFP DC cPARP 24h

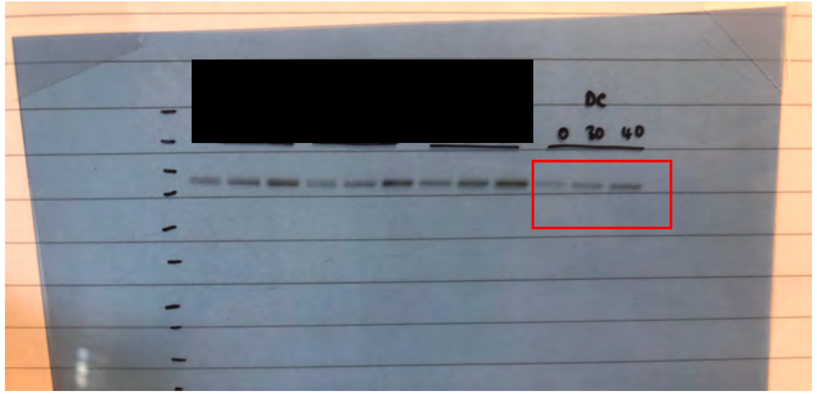

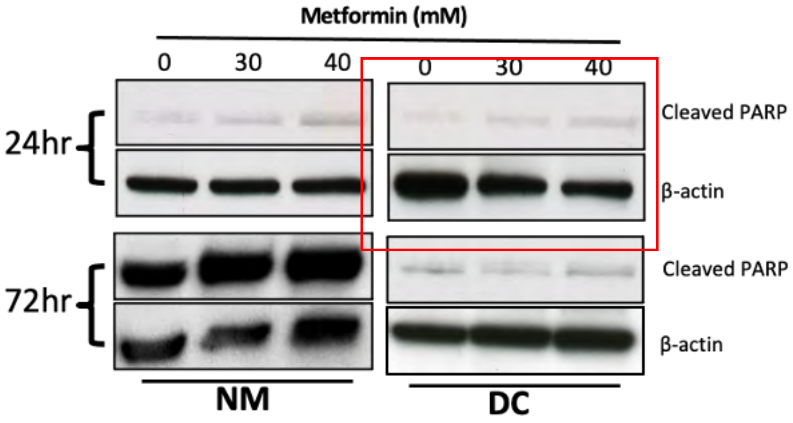

Figure 1G p21

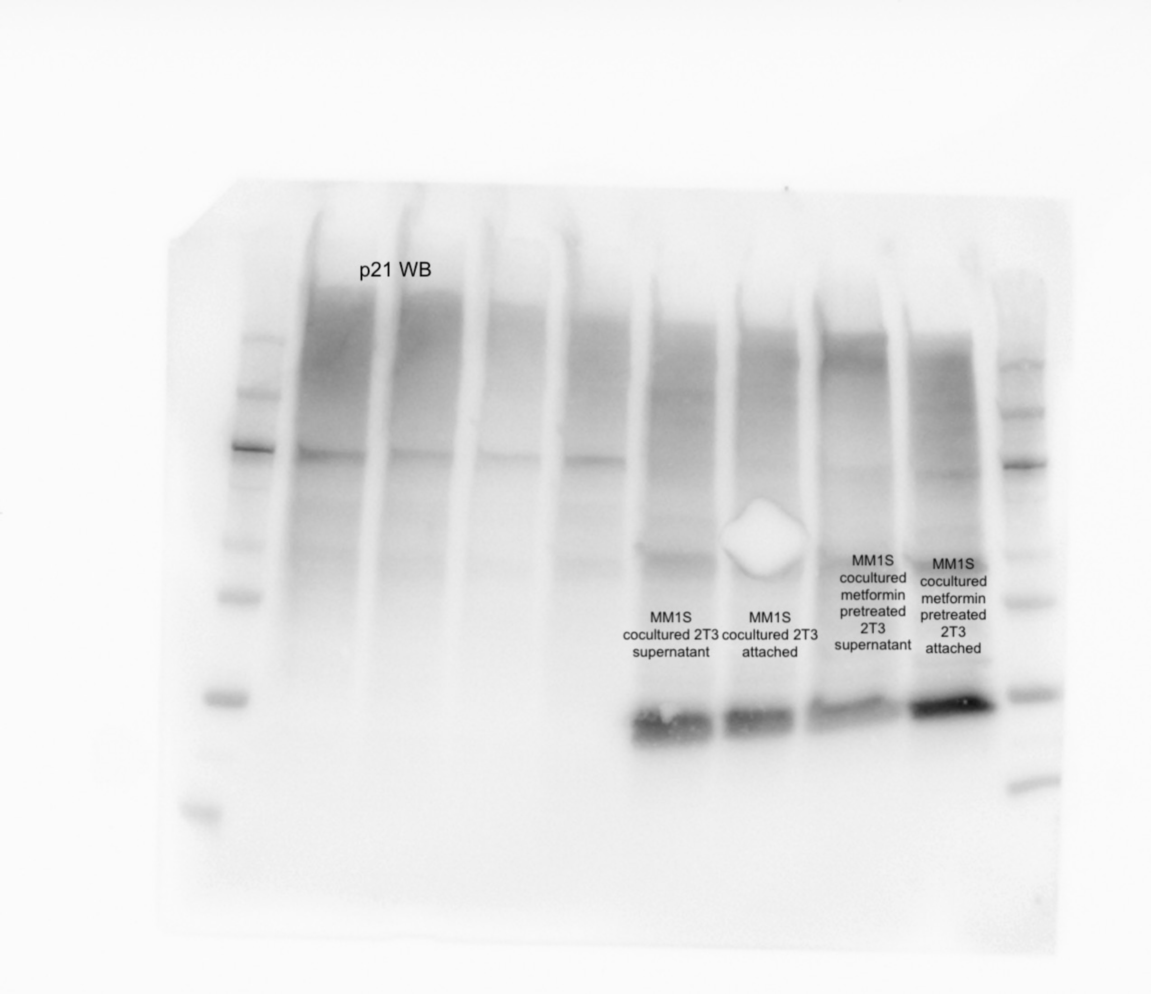

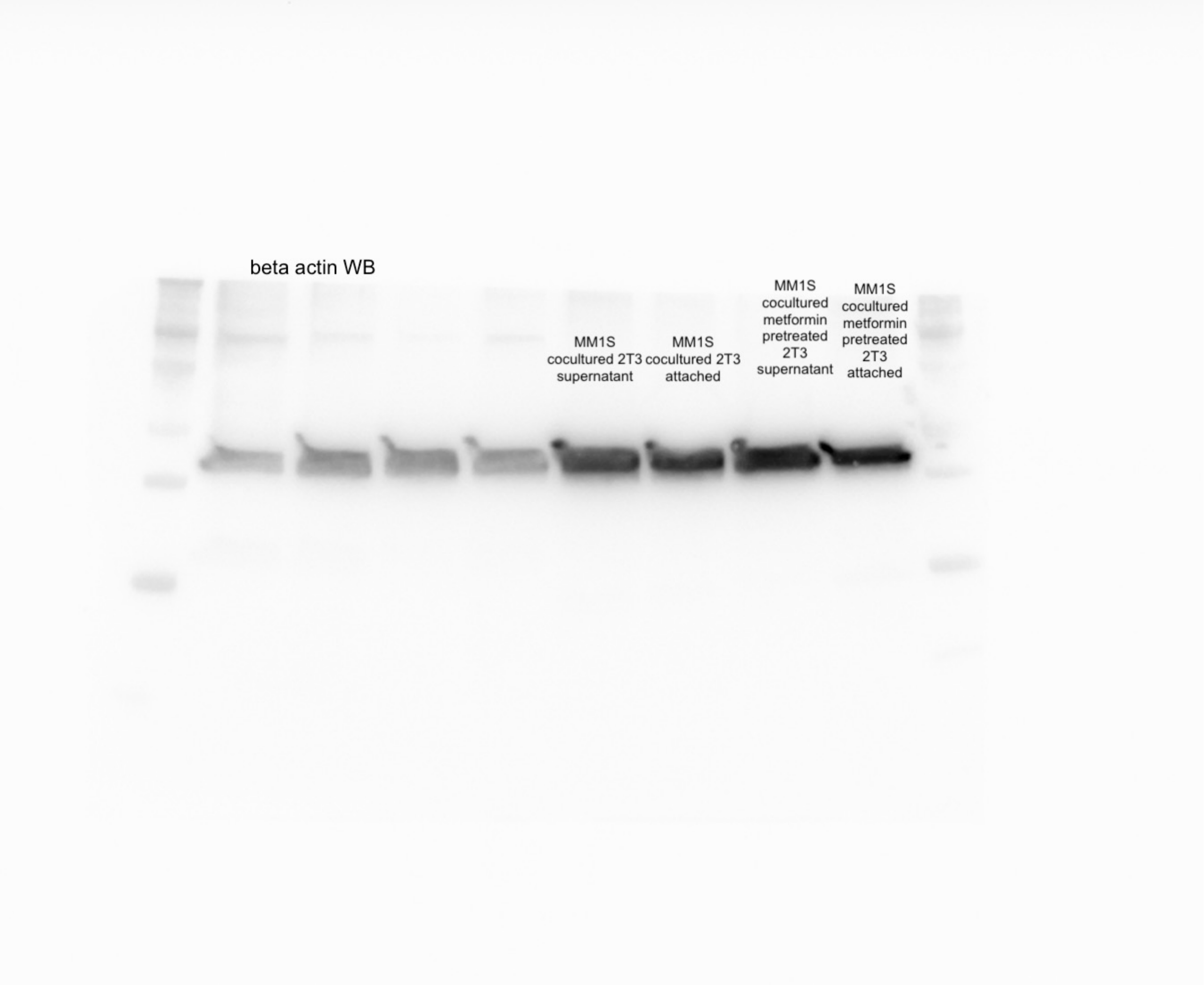

Figure 2B (2T3)

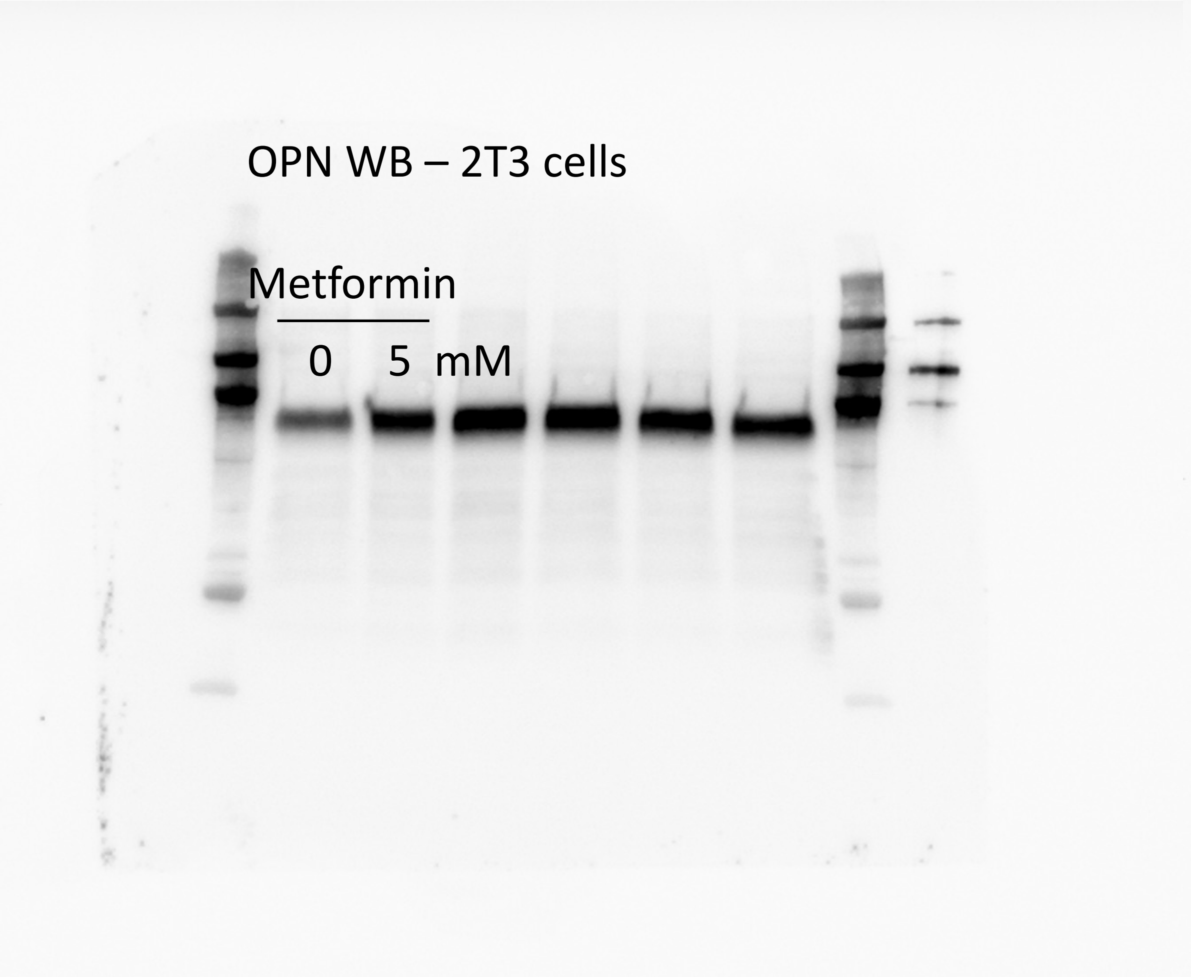

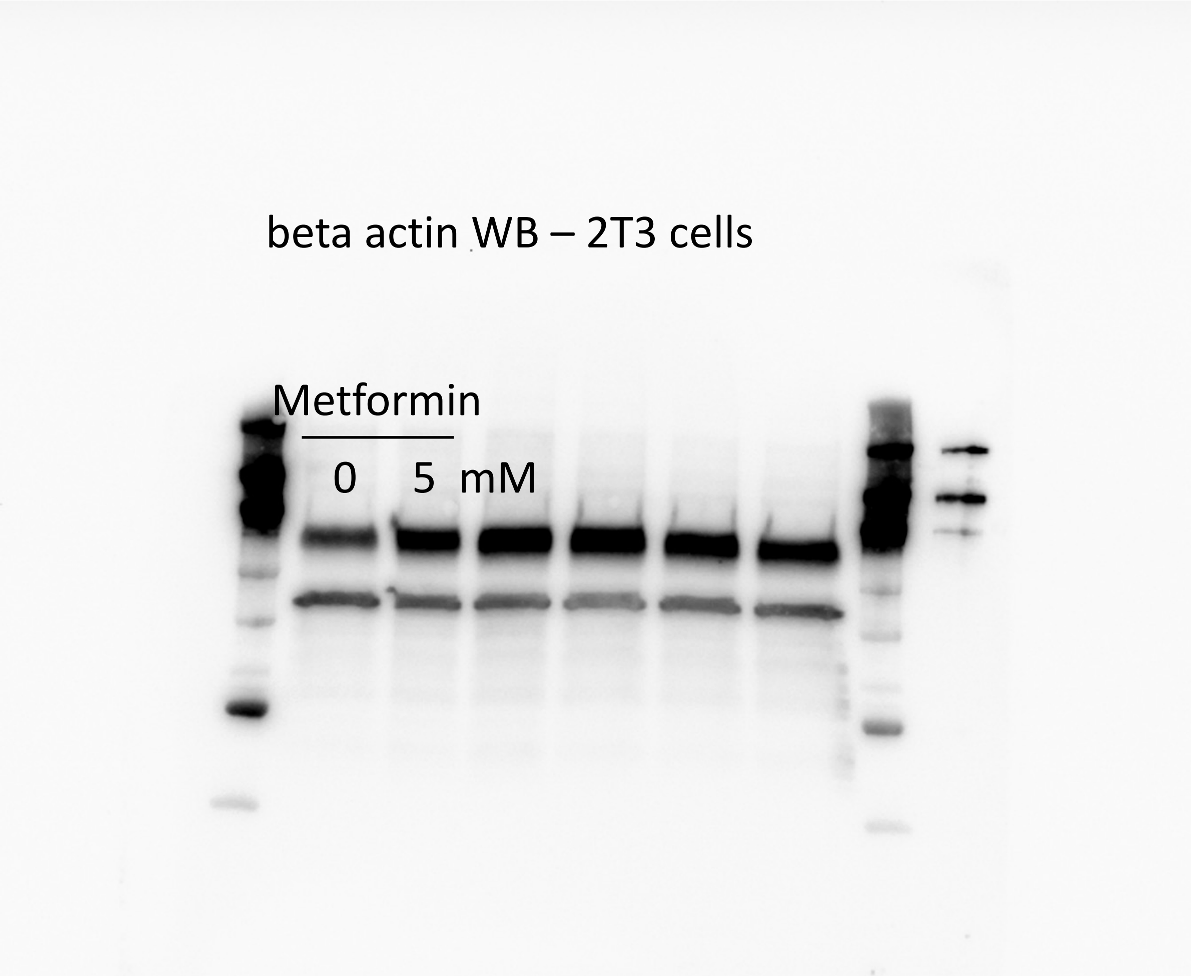

Figure 2B (ST2)

ST2 48 metformin - OPN

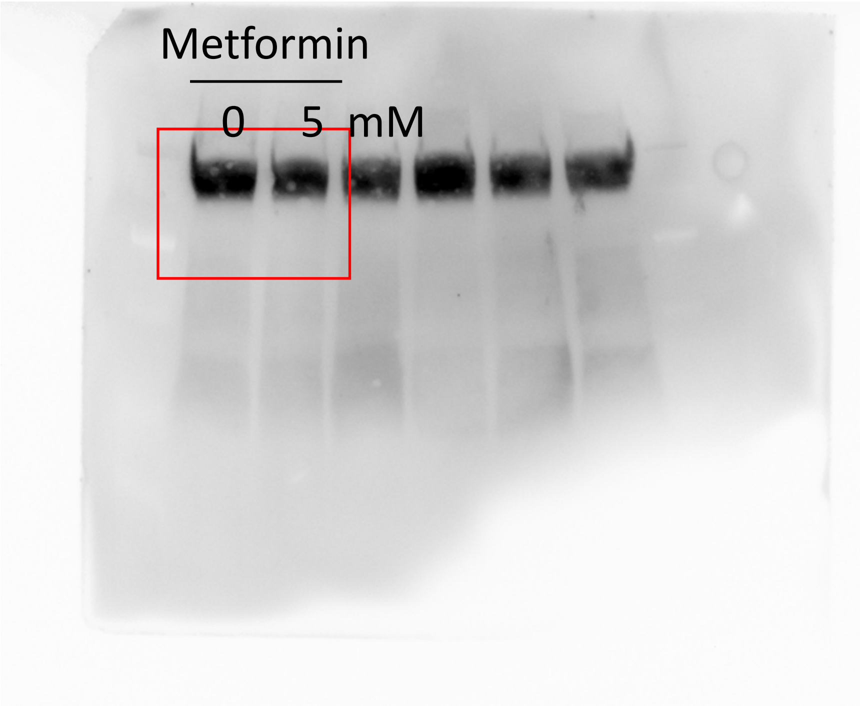

ST2 48 metformin – beta actin

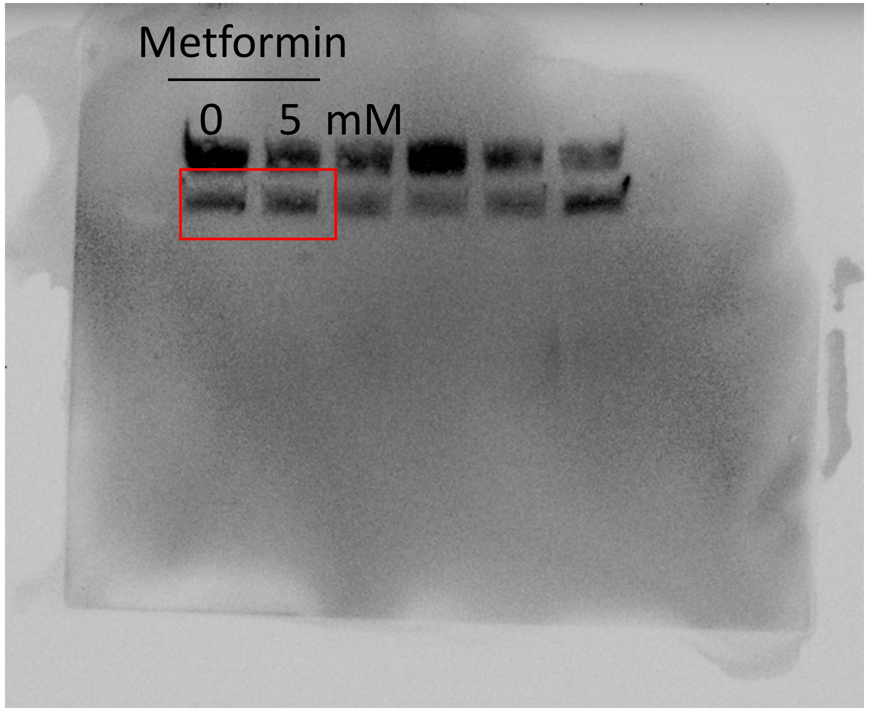

Figure 2D OPN

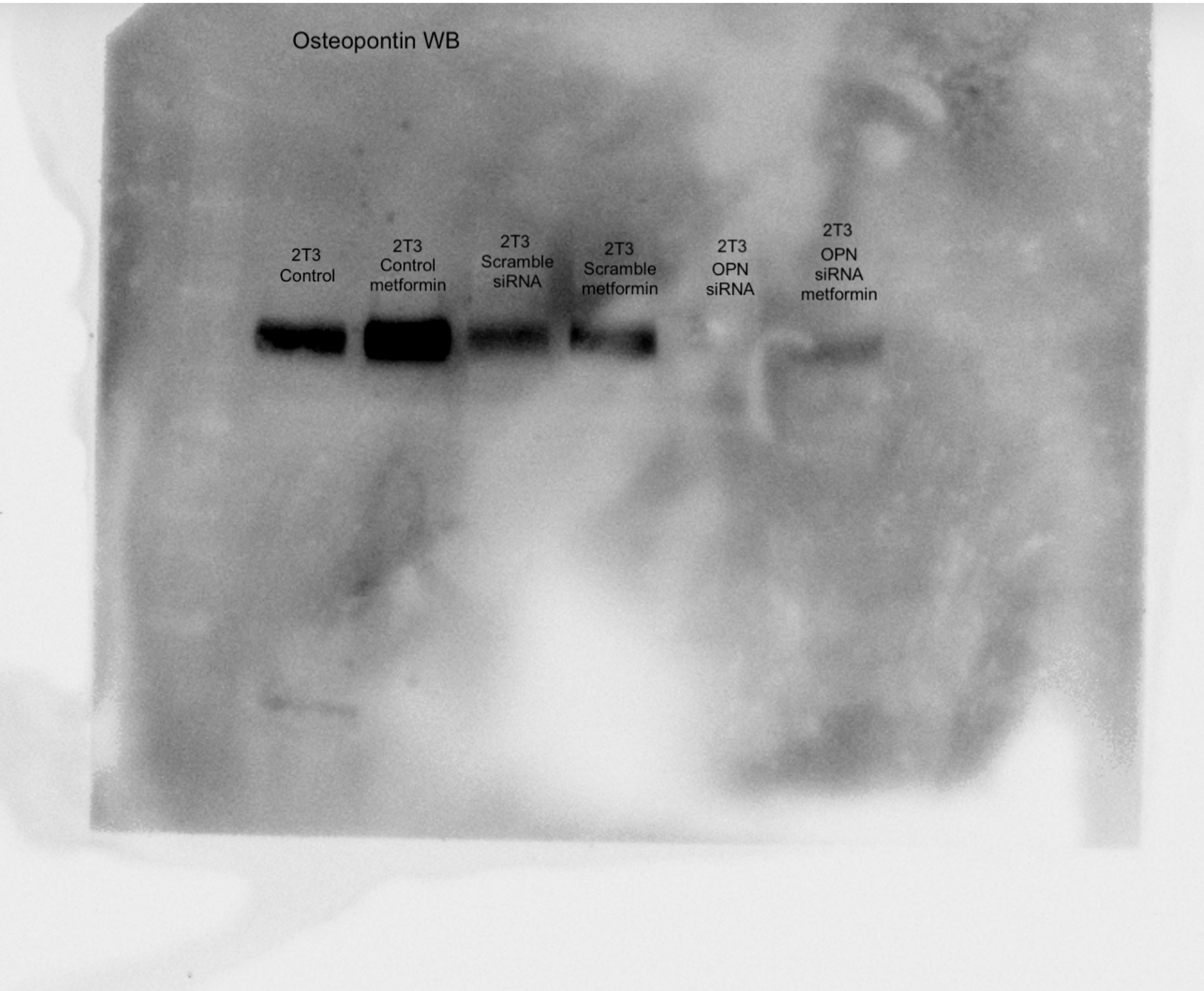

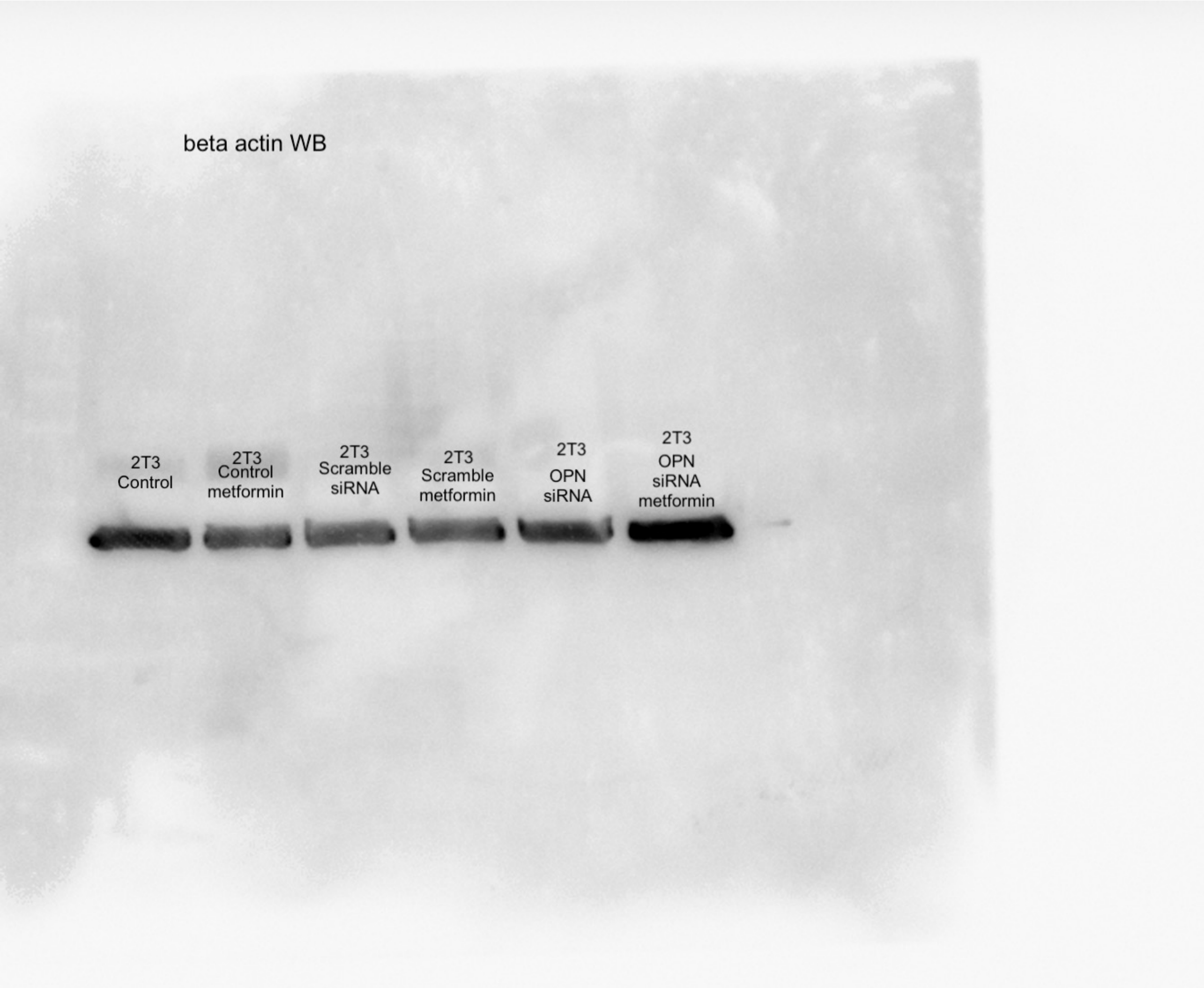

Supplement: Supplementary file 1 [file mmc1.zip › WB originals.pdf]
